# An integrated privacy preserving data aggregation framework for IoT networks using homomorphic encryption and secure computation

**DOI:** 10.1038/s41598-026-48831-6

**Published:** 2026-04-28

**Authors:** Qiang Qin, Yongjiao Yang, Jiaxin Lin, Hanye Huang, Yuetian Huang

**Affiliations:** Guangdong Power Grid Co., Ltd., Guangzhou, 510220 Guangdong China

**Keywords:** Internet of things, Privacy-preserving aggregation, Homomorphic encryption, Differential privacy, Secure multi-party computation, Data security, Engineering, Mathematics and computing

## Abstract

The widespread adoption of Internet of Things (IoT) devices has opened new possibilities for data-driven decision making while simultaneously raising serious concerns about the protection of sensitive personal information. This paper presents an integrated privacy-preserving data aggregation framework that strategically combines homomorphic encryption, secure multi-party computation, and differential privacy mechanisms. We design a hierarchical three-tier protocol architecture in which IoT devices encrypt their measurements using Paillier homomorphic encryption, edge nodes carry out secure aggregation through distributed computation, and cloud servers inject calibrated differential privacy noise prior to threshold-based decryption. The framework reduces reliance on trusted third parties through Shamir secret sharing while striving to maintain computational efficiency appropriate for resource-constrained devices. Experimental evaluation involving 1000 simulated IoT devices across 30 independent trials demonstrates that the protocol completes aggregation in 3.82 ± 0.35 s (mean ± standard deviation) with 2.8% ± 0.6% relative error under moderate privacy budgets (ε = 1.0, 95% CI: [2.4%, 3.2%]). Security analysis under the semi-honest adversary model indicates that the protocol satisfies semantic security assumptions and ε-differential privacy guarantees. These results suggest that meaningful privacy protection can be achieved while preserving practical utility, offering a potential solution for privacy-sensitive IoT deployments in smart cities, healthcare monitoring, and industrial applications, although certain limitations regarding scalability and adversary models require further investigation.

**Supplementary Information:**

The online version contains supplementary material available at 10.1038/s41598-026-48831-6.

## Introduction

The proliferation of Internet of Things (IoT) devices has fundamentally transformed how we collect, process, and analyze data across diverse domains, ranging from smart cities to healthcare monitoring systems^1^. These interconnected devices generate massive volumes of sensitive information continuously, creating both unprecedented opportunities for data-driven insights and significant privacy concerns that demand immediate attention^2^. As IoT deployments expand to encompass millions of edge devices, the challenge of aggregating data while preserving individual privacy has emerged as a critical bottleneck in realizing the full potential of IoT ecosystems.

Traditional data aggregation approaches in IoT networks typically rely on centralized architectures where raw data streams directly to a central server for processing^3^. This paradigm, however, introduces severe vulnerabilities: the aggregation server becomes a single point of failure and an attractive target for adversaries seeking access to sensitive personal information. Recent high-profile data breaches have demonstrated that even well-protected centralized systems remain susceptible to sophisticated attacks, raising fundamental questions about whether we can continue trusting centralized entities with our most sensitive data^4^. Moreover, regulatory frameworks such as GDPR and CCPA impose stringent requirements on data collection and processing, compelling researchers to reconsider how IoT systems handle personal information from the ground up.

Current research efforts have explored various cryptographic primitives to address these privacy challenges. Homomorphic encryption has attracted considerable interest because it enables computation on encrypted data without requiring decryption, thereby maintaining confidentiality throughout the processing pipeline^5^. Secure multi-party computation offers another promising direction by allowing multiple parties to jointly compute functions over their private inputs while ensuring that no party learns anything beyond the final result^6^. Differential privacy has also gained traction as a rigorous mathematical framework for quantifying and limiting privacy loss in data analysis^7^. Despite these advances, existing solutions often treat these techniques in isolation, failing to address the holistic requirements of practical IoT deployments.

Several fundamental challenges continue to impede the widespread adoption of privacy-preserving IoT data aggregation. First, computational efficiency remains a persistent concern: homomorphic encryption operations introduce substantial computational overhead that can overwhelm resource-constrained IoT devices^8^. Second, achieving meaningful privacy guarantees while maintaining data utility presents inherent trade-offs that current methods struggle to balance effectively. Third, existing protocols often assume static network topologies and fail to accommodate the dynamic nature of real-world IoT deployments where devices frequently join or leave the network. Fourth, scalability becomes problematic when the number of participating devices grows beyond a few hundred, as communication and computation costs increase dramatically^9^.

This research becomes particularly necessary when we recognize that IoT applications increasingly involve sensitive personal data—health records, location traces, consumption patterns—that could cause significant harm if exposed or misused. The significance of developing robust privacy-preserving aggregation protocols extends beyond technical considerations to encompass ethical responsibilities and societal trust in emerging technologies. Without effective solutions, we risk either abandoning valuable IoT applications due to privacy concerns or proceeding with insufficient protections that expose individuals to harm^10^. Furthermore, as IoT systems become embedded in critical infrastructure, ensuring both data privacy and system reliability becomes not merely desirable but essential for maintaining public safety and confidence.

The present work addresses these challenges by proposing an integrated framework that strategically combines homomorphic encryption, secure multi-party computation, and differential privacy to achieve efficient and privacy-preserving data aggregation in IoT networks. Unlike existing approaches that typically apply these techniques in isolation, our unified protocol exploits their complementary strengths while mitigating individual limitations. For instance, SecAgg and its variants rely primarily on secret sharing without incorporating formal differential privacy guarantees^11^, whereas pure differential privacy schemes often suffer from excessive noise accumulation in distributed settings. Our approach differs from these methods in several important ways. We design a hybrid encryption scheme that strategically applies homomorphic operations only where necessary, substantially reducing computational burden on edge devices compared to fully homomorphic encryption approaches. We develop a distributed secure computation protocol that reduces the need for trusted third parties while remaining resilient to dynamic network changes. We integrate differential privacy mechanisms directly into the aggregation process at both local and global levels, providing formal privacy guarantees with better utility than purely local approaches. We also propose an adaptive parameter tuning strategy that automatically adjusts privacy-utility trade-offs based on application requirements and network conditions.

These contributions advance the current understanding of privacy-preserving IoT data aggregation by suggesting that meaningful privacy protection can be achieved without entirely sacrificing practical efficiency. Our framework provides a foundation for deploying IoT systems that respect individual privacy while delivering the data insights necessary for informed decision-making across critical applications, though we acknowledge that further validation across diverse real-world scenarios remains necessary.

## Related theory and technical foundations

### Homomorphic encryption technology

Homomorphic encryption represents a transformative cryptographic paradigm that permits meaningful computations on ciphertext without requiring decryption, thus preserving data confidentiality throughout the entire processing lifecycle^12^. This capability addresses a long-standing challenge in cryptography: how can we process sensitive data while keeping it encrypted? The fundamental property that distinguishes homomorphic encryption from conventional encryption schemes lies in its algebraic structure, which preserves specific operations when transitioning from plaintext to ciphertext domains^13^.

We can categorize homomorphic encryption schemes based on the types and quantities of operations they support. Partially homomorphic encryption (PHE) allows unlimited operations of a single type—either addition or multiplication—on encrypted data^14^. The Paillier cryptosystem exemplifies this category by supporting additive homomorphism, enabling the computation of encrypted sums without decryption. For two ciphertexts $\:E\left({m}_{1}\right)$ and $\:E\left({m}_{2}\right)$, Paillier encryption satisfies the following standard homomorphic property:

1 $$\:E\left({m}_{1}\right)\times\:E\left({m}_{2}\right)=E({m}_{1}+{m}_{2})\mathrm{p}\mathrm{m}\mathrm{o}\mathrm{d}{n}^{2}$$

where $\:n$ represents the RSA modulus^15^. This additive property makes Paillier particularly suitable for privacy-preserving data aggregation scenarios where we need to compute encrypted sums across multiple data sources.

Fully homomorphic encryption (FHE), in contrast, supports arbitrary computations on encrypted data by enabling both addition and multiplication operations simultaneously^16^. Gentry’s breakthrough work in 2009 demonstrated the theoretical feasibility of FHE, though practical implementations remained computationally prohibitive for years. Contemporary FHE schemes such as BGV (Brakerski-Gentry-Vaikuntanathan) and BFV (Brakerski-Fan-Vercauteren) have substantially improved efficiency through clever noise management techniques^17^. These schemes build upon the Ring Learning With Errors (RLWE) problem, which provides both security guarantees and computational efficiency. The BGV scheme performs homomorphic operations within polynomial rings, where a ciphertext $\:c$ encrypting message $\:m$ satisfies:

2 $$c = [a \cdot s + e + m]_{q}$$

with $\:s$ denoting the secret key, $\:a$ a random polynomial, $\:e$ a small error term, and $\:q$ the ciphertext modulus (standard RLWE encryption form)^18^.

The mathematical foundation of these schemes rests on computationally hard problems from lattice-based cryptography. RLWE’s hardness assumption provides security against quantum attacks, distinguishing homomorphic encryption from RSA-based systems that remain vulnerable to quantum algorithms^19^. However, noise accumulation during homomorphic operations poses a fundamental challenge: each computation incrementally increases the error term embedded in ciphertexts. When noise exceeds a threshold, decryption fails. The BFV scheme addresses this through a scale-invariant approach where the noise growth rate remains independent of plaintext modulus:

3 $$\:\mathrm{N}\mathrm{o}\mathrm{i}\mathrm{s}\mathrm{e}({c}_{1}\odot\:{c}_{2})\approx\:\mathrm{N}\mathrm{o}\mathrm{i}\mathrm{s}\mathrm{e}\left({c}_{1}\right)+\mathrm{N}\mathrm{o}\mathrm{i}\mathrm{s}\mathrm{e}\left({c}_{2}\right)+\varDelta\:$$

where $\:\odot\:$ represents homomorphic multiplication and $\:\varDelta\:$ denotes additional noise (standard noise growth formula)^20^. This controlled noise behavior enables deeper circuit evaluation compared to earlier FHE constructions. However, even with these improvements, FHE remains computationally intensive for IoT applications, which motivates our choice of the more efficient Paillier partially homomorphic encryption scheme for aggregation tasks where only additive operations are required.

### Secure multi-party computation technology

Secure multi-party computation (MPC) provides a cryptographic framework enabling multiple parties to jointly compute a function over their private inputs while ensuring that no participant learns anything beyond the designated output^21^. The core challenge MPC addresses is deceptively simple yet profoundly important: can mutually distrusting parties collaborate on computations without revealing their individual data? Yao’s seminal work in the 1980s demonstrated that such protocols are theoretically possible, though practical implementations have required decades of refinement^22^.

Security models for MPC typically distinguish between semi-honest and malicious adversaries. Semi-honest participants follow protocol specifications but may attempt to infer additional information from received messages^23^. Malicious adversaries, conversely, can deviate arbitrarily from prescribed protocols. Most practical MPC systems target semi-honest security due to the substantial overhead associated with malicious security guarantees. The security definition commonly relies on the simulation paradigm: a protocol securely computes function $\:f$ if whatever an adversary learns from protocol execution could have been simulated given only the adversary’s input and output^24^.

Secret sharing forms a fundamental building block for MPC protocols. Shamir’s secret sharing scheme splits a secret $\:s$ into $\:n$ shares such that any $\:t$ shares can reconstruct the secret, yet fewer than $\:t$ shares reveal nothing (standard $\:(t,n)$-threshold secret sharing)^25^. The reconstruction formula follows from Lagrange interpolation:

4 $$\:s=\sum\:_{i=1}^{t}{s}_{i}\prod\:_{j=1,j\ne\:i}^{t}\frac{{x}_{j}}{{x}_{j}-{x}_{i}}$$

where $\:{s}_{i}$ represents individual shares and $\:{x}_{i}$ denotes evaluation points. This mathematical property enables distributed computation: parties can perform operations on shares locally, then combine results to obtain the output.

Oblivious transfer (OT) constitutes another essential primitive, particularly in two-party computation scenarios. In 1-out-of-2 OT, a sender possesses two messages $\:{m}_{0}$ and $\:{m}_{1}$, while a receiver wishes to obtain $\:{m}_{b}$ for choice bit $\:b$ without revealing $\:b$ to the sender^26^. The sender simultaneously learns nothing about which message was received. Modern OT protocols build upon public-key cryptography, though OT extension techniques dramatically reduce computational costs by generating many OTs from a small number of base OTs.

Garbled circuits, introduced by Yao, offer an elegant approach to two-party computation. The circuit constructor (garbler) encrypts a Boolean circuit representing the target function, assigning random labels to each wire^27^. The evaluator then obliviously obtains labels corresponding to their input bits through OT, evaluates the garbled circuit gate-by-gate, and learns the output. For a gate computing $\:g(a,b)=c$, the garbling process generates:

5 $$\:{\mathrm{E}\mathrm{n}\mathrm{c}}_{{k}_{a}^{0},{k}_{b}^{0}}\left({k}_{c}^{g\left(\mathrm{0,0}\right)}\right),{\mathrm{E}\mathrm{n}\mathrm{c}}_{{k}_{a}^{0},{k}_{b}^{1}}\left({k}_{c}^{g\left(\mathrm{0,1}\right)}\right),{\mathrm{E}\mathrm{n}\mathrm{c}}_{{k}_{a}^{1},{k}_{b}^{0}}\left({k}_{c}^{g\left(\mathrm{1,0}\right)}\right),{\mathrm{E}\mathrm{n}\mathrm{c}}_{{k}_{a}^{1},{k}_{b}^{1}}\left({k}_{c}^{g\left(\mathrm{1,1}\right)}\right)$$

where $\:{k}_{w}^{v}$ denotes the label for wire $\:w$ carrying value $\:v$ (standard garbled gate construction)^28^.

In data aggregation contexts, MPC enables privacy-preserving statistics computation across distributed datasets. IoT nodes can collectively compute aggregate functions—sums, averages, variances—without exposing individual measurements to any single entity, including the aggregation server. This property proves particularly valuable when data sources belong to competing organizations or when regulatory constraints prohibit raw data sharing.

### Differential privacy theory

Differential privacy emerged as a rigorous mathematical framework for quantifying privacy guarantees in statistical data release, addressing fundamental limitations of earlier anonymization approaches^29^. The definition captures an intuitive notion: query results should appear nearly identical whether any individual’s data is included or excluded from the dataset. More formally, a randomized mechanism $\:\mathcal{M}$ satisfies $\:\epsilon\:$-differential privacy if for all neighboring datasets $\:D$ and $\:{D}^{{\prime\:}}$ differing in a single record, and for all possible output sets $\:S$ (standard differential privacy definition):

6 $$\Pr \left[ {{\mathcal{M}}\left( D \right) \in S} \right] \le e^{\varepsilon } \cdot \Pr \left[ {{\mathcal{M}}\left( {D^{\prime}} \right) \in S} \right]$$

where $\:\epsilon\:$ represents the privacy budget parameter^30^. Smaller $\:\epsilon\:$ values provide stronger privacy guarantees but typically reduce data utility—a fundamental trade-off inherent to privacy-preserving data analysis.

The privacy budget $\:\epsilon\:$ quantifies the maximum privacy loss an individual faces by participating in the computation. When $\:\epsilon\:$ approaches zero, outputs become essentially independent of any single record, offering robust privacy protection. Conversely, larger $\:\epsilon\:$ values permit greater information leakage but enable more accurate query responses^31^. Privacy budgets compose across multiple queries: executing $\:k$ queries each satisfying $\:\epsilon\:$-differential privacy yields overall privacy guarantee of $\:k\epsilon\:$, highlighting the importance of careful budget al.location in practice.

We distinguish between two deployment models: global and local differential privacy. Global differential privacy assumes a trusted curator who accesses raw data, adds calibrated noise, and releases sanitized results^32^. This centralized model achieves better accuracy for a given privacy level but requires trusting the curator with unprotected data. Local differential privacy eliminates this trust assumption by requiring each data provider to perturb their own data before sharing it^33^. While local models align better with privacy principles and regulatory requirements, they suffer from substantially degraded accuracy due to noise accumulation across distributed randomization.

Noise addition mechanisms implement differential privacy by injecting carefully calibrated randomness into query outputs. The Laplace mechanism adds noise drawn from a Laplace distribution with scale proportional to the query’s sensitivity—the maximum change in query output when modifying a single record^34^. For a query $\:f$ with sensitivity $\:\varDelta\:f$, the Laplace mechanism releases:

7 $$\:\mathcal{M}\left(D\right)=f\left(D\right)+\mathrm{L}\mathrm{a}\mathrm{p}\left(\frac{\varDelta\:f}{\epsilon\:}\right)$$

where $\:\mathrm{L}\mathrm{a}\mathrm{p}\left(\lambda\:\right)$ denotes the Laplace distribution with scale parameter $\:\lambda\:$ (standard Laplace mechanism)^35^. The Gaussian mechanism serves as an alternative for approximate differential privacy, offering computational advantages in certain scenarios but requiring careful parameter tuning to achieve desired privacy levels.

Comparing differential privacy with alternative privacy-preserving techniques reveals distinct characteristics. Unlike k-anonymity or l-diversity, which rely on syntactic transformations and have known vulnerabilities to background knowledge attacks, differential privacy provides semantic guarantees that remain robust against arbitrary auxiliary information^36^. Cryptographic approaches like homomorphic encryption protect data during computation but do not inherently limit information leakage from outputs—differential privacy complements these techniques by bounding what query results reveal about individuals. This complementary nature motivates hybrid approaches that combine differential privacy with cryptographic protections to achieve comprehensive privacy guarantees.

## Design of data aggregation protocol based on homomorphic encryption and secure multi-party computation

### System model and threat model

**Fig. 1:**
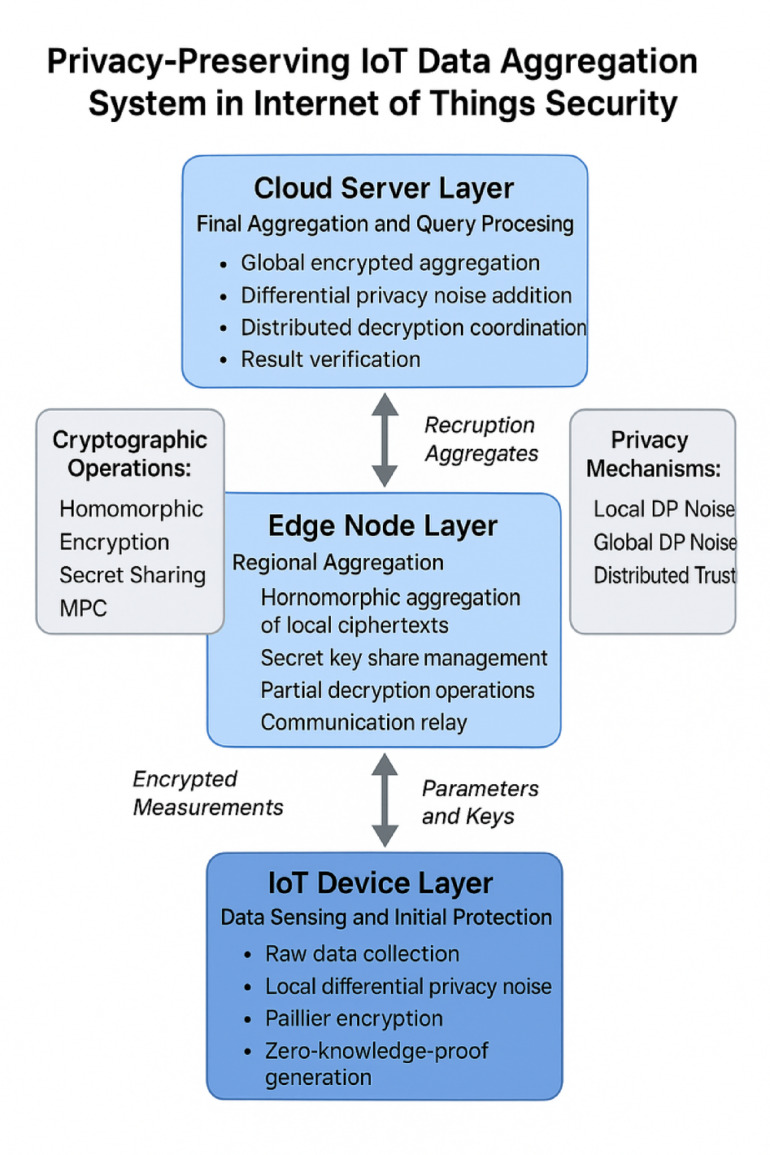
System architecture for privacy-preserving IoT data aggregation.

Our proposed data aggregation framework operates within a three-tier hierarchical architecture that reflects contemporary IoT deployment patterns while addressing their inherent security challenges. Figure 1 illustrates the overall system architecture, depicting the interaction flows among participating entities and the cryptographic operations performed at each tier. The architecture consists of IoT devices at the sensing layer, edge nodes providing intermediate computation capabilities, and cloud servers delivering centralized aggregation services.

As shown in Fig. 1, the three-tier structure enables distributed processing while maintaining privacy guarantees throughout the aggregation pipeline. IoT devices constitute the foundational layer, encompassing resource-constrained sensors and actuators that generate raw measurements from physical environments^37^. These devices possess limited computational power and battery capacity, constraining the cryptographic operations they can perform locally. Edge nodes occupy the intermediate layer, providing enhanced computational resources and serving as aggregation points for geographically proximate IoT devices. Cloud servers represent the top tier, offering substantial storage and processing capabilities for final data aggregation and analytics.

Table 1 summarizes the distinct roles and functional capabilities of each system entity. As presented in Table 1, we observe significant variations in computational capacity, communication overhead, and security responsibilities across the three entity types. IoT devices handle initial data collection and lightweight encryption, while edge nodes perform intermediate aggregation using homomorphic properties. Cloud servers conduct final aggregation and respond to analytical queries, though they operate on encrypted data without accessing plaintext values.

**Table 1:** System entity roles and functions comparison.

Entity type	Computational capacity	Primary functions	Security responsibilities	Communication patterns
IoT Device	Limited (constrained)	Data sensing, local encryption, differential privacy noise addition	Input data protection, local privacy preservation	Periodic uplink to edge nodes
Edge Node	Moderate (intermediate)	Regional aggregation, partial decryption, secret share generation	Secure computation coordination, aggregate verification	Bidirectional with devices and cloud
Cloud Server	High (powerful)	Global aggregation, encrypted query processing, result distribution	System-wide security maintenance, access control	Downlink to edge nodes and end users
Trusted Authority	High (offline)	Key generation, parameter initialization, dispute resolution	Cryptographic key management, system bootstrap	Limited interaction during setup phase

The communication model follows a bottom-up aggregation pattern where data flows from devices through edge nodes to cloud servers^38^. Devices transmit encrypted measurements to their designated edge nodes at regular intervals or upon specific trigger events. Edge nodes aggregate received ciphertexts homomorphically, then forward intermediate results to cloud servers. This hierarchical communication structure reduces network congestion and distributes computational burden across system tiers.

We establish our threat model under the honest-but-curious (semi-honest) adversarial assumption, where entities follow protocol specifications correctly but may attempt to infer sensitive information from observed data^39^. This assumption represents a practical middle ground commonly adopted in secure computation research, though it does carry important limitations. Concretely, the semi-honest boundary means that we assume all entities execute each protocol step exactly as specified—transmitting correctly formatted ciphertexts, generating valid secret shares, and applying prescribed noise distributions—while potentially recording and analyzing all messages they observe. Specifically, semi-honest adversaries do not deviate from the protocol by sending malformed messages or refusing to participate, which may not fully capture real-world attack scenarios where adversaries behave maliciously. Cloud servers represent potential adversaries that could analyze aggregated ciphertexts or collude with compromised edge nodes. We assume adversaries cannot break underlying cryptographic primitives but may exploit protocol weaknesses or side-channel information. External attackers might eavesdrop on communication channels, inject false data, or attempt to link anonymized measurements to specific devices. Crucially, we do not assume any fully trusted third party beyond the initial system setup phase.

Regarding our choice of Paillier encryption, we selected this scheme over alternatives such as BGV or BFV fully homomorphic encryption for several practical reasons. Paillier encryption offers additive homomorphism sufficient for aggregation tasks while requiring significantly less computational overhead than lattice-based FHE schemes^40^. Our preliminary benchmarks on Raspberry Pi 4 devices indicated that Paillier encryption completes in approximately 35 ms per operation, whereas even optimized FHE implementations require seconds per operation on similar hardware. Additionally, Paillier’s semantic security under the decisional composite residuosity assumption provides well-understood security guarantees^12^. We acknowledge that Paillier encryption still imposes non-trivial overhead on extremely resource-constrained devices such as low-end microcontrollers, and future work should explore lightweight alternatives for such deployment scenarios^41^.

Our security objectives encompass multiple dimensions of protection. Data confidentiality requires that raw measurements remain hidden from all entities except authorized data owners. Privacy preservation ensures that aggregated results do not reveal individual contributions beyond what differential privacy permits. Integrity guarantees detect any unauthorized modifications to data during transmission or aggregation. Authentication mechanisms verify that only legitimate devices participate in the aggregation process^42^. These objectives collectively define our design requirements: the protocol must achieve strong privacy guarantees while maintaining computational efficiency suitable for resource-constrained IoT environments, resist collusion attacks among multiple compromised entities, and provide verifiable correctness for aggregated results.

### Core protocol mechanism design

**Fig. 2:**
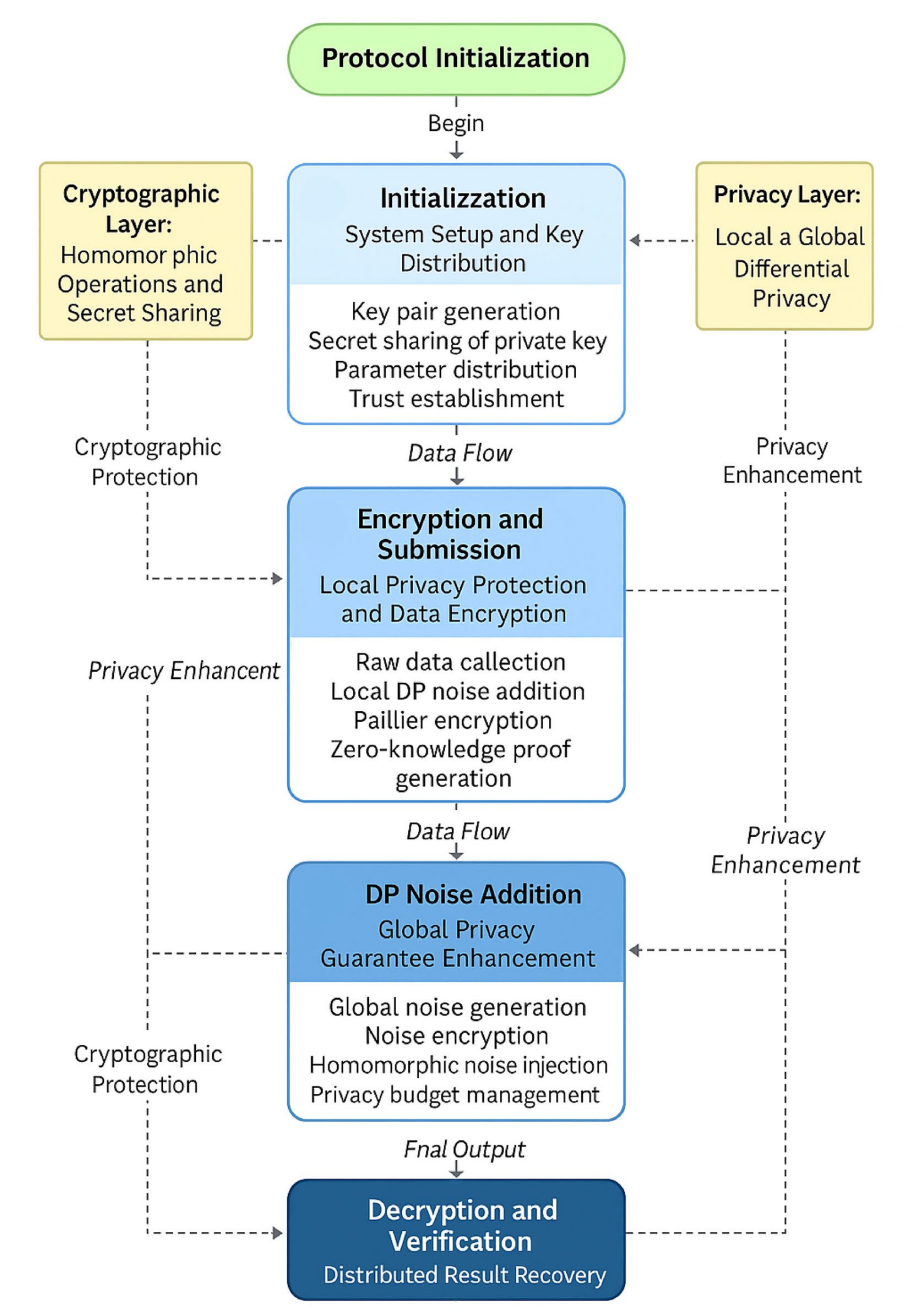
Protocol execution workflow across five phases.

Our protocol executes through five distinct phases that orchestrate cryptographic operations across the three-tier architecture. Figure 2 depicts the complete protocol execution flow, showing how data transitions through encryption, aggregation, noise addition, and decryption stages while maintaining privacy guarantees at each step.

The initialization phase establishes cryptographic parameters and distributes keys among participating entities. As shown in Fig. 2, this phase precedes all data collection activities and occurs only once during system deployment. The trusted authority generates a public-private key pair $\:(pk,sk)$ for the Paillier cryptosystem and distributes $\:pk$ to all IoT devices while partitioning $\:sk$ into shares using Shamir’s secret sharing scheme^43^. Each edge node receives a share $\:s{k}_{i}$ such that any threshold number of shares can reconstruct the complete secret key. This distribution prevents any single entity from independently decrypting aggregated data. Additionally, devices receive differential privacy parameters $\:(\epsilon\:,\delta\:)$ that govern noise calibration in subsequent phases.

**Fig. 3:**
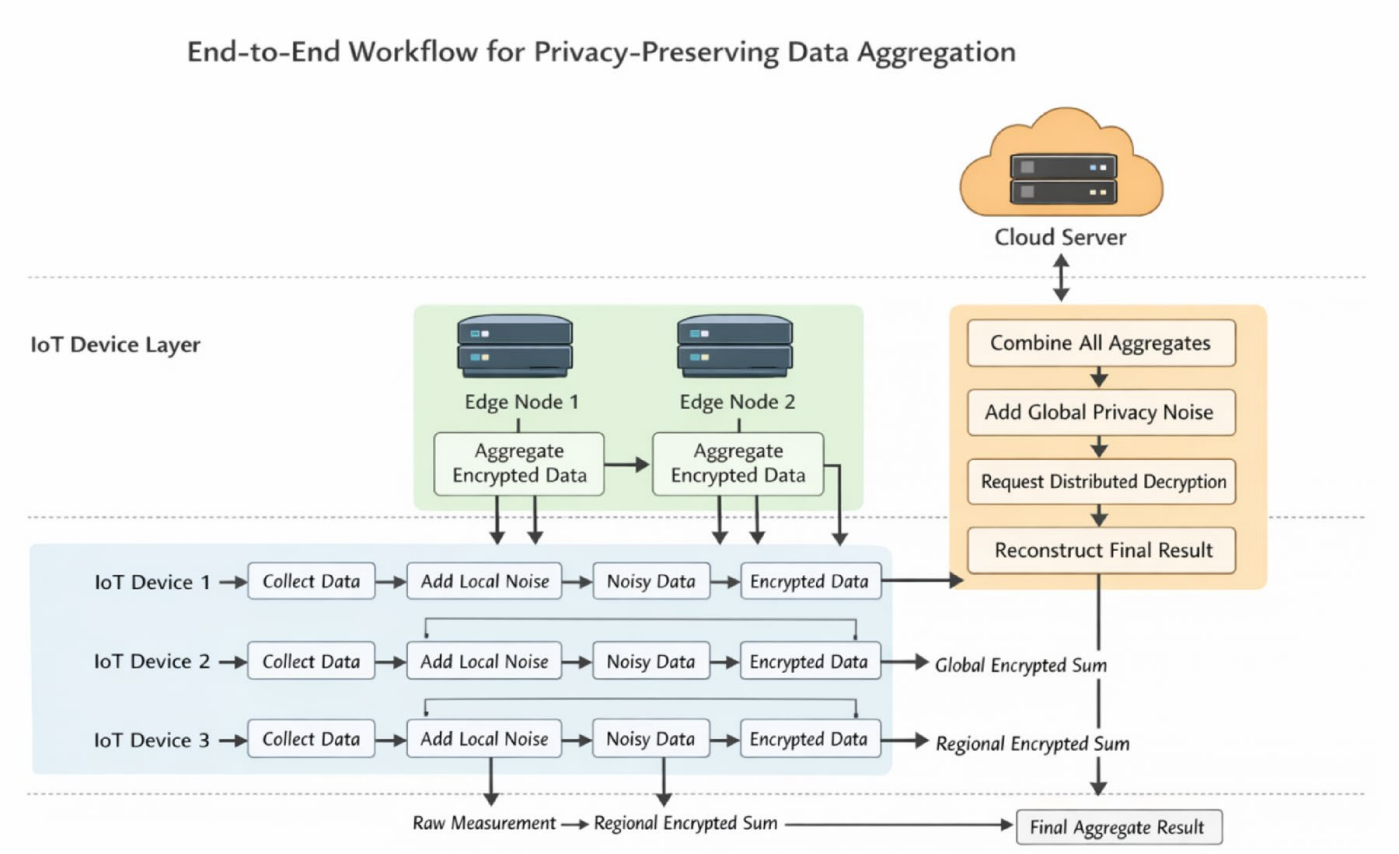
End-to-end workflow diagram for privacy-preserving data aggregation.

Figure 3 illustrates the complete end-to-end workflow of our privacy-preserving data aggregation framework. As shown in Fig. 3, raw measurements collected by IoT devices first undergo local noise injection for differential privacy protection, followed by Paillier encryption before transmission to edge nodes. The edge nodes then perform homomorphic aggregation on the received ciphertexts and forward regional encrypted sums to the cloud server. The cloud server combines all regional aggregates, adds global privacy noise, and initiates the distributed decryption process by requesting partial decryption shares from threshold-many edge nodes. Finally, the cloud server reconstructs the privacy-protected aggregate result through Lagrange interpolation, ensuring that no single entity can access individual measurements throughout the entire process.

During the data encryption and submission phase, IoT devices collect measurements and apply local privacy protections before transmission. Device $\:i$ with raw measurement $\:{m}_{i}$ first adds calibrated Laplace noise to achieve local differential privacy:

8 $$\:{\stackrel{\sim}{m}}_{i}={m}_{i}+\mathrm{L}\mathrm{a}\mathrm{p}\left(\frac{\varDelta\:f}{{\epsilon\:}_{local}}\right)$$

where $\:\varDelta\:f$ denotes the sensitivity of the measurement function and $\:{\epsilon\:}_{local}$ represents the allocated local privacy budget. The device then encrypts the perturbed value using the Paillier public key: $\:{c}_{i}={E}_{pk}\left({\stackrel{\sim}{m}}_{i}\right)$^44^. This ciphertext gets transmitted to the designated edge node along with a zero-knowledge proof demonstrating that the encrypted value falls within the valid measurement range, preventing pollution attacks from malicious devices.

The secure multi-party computation aggregation phase performs hierarchical aggregation while maintaining ciphertext form throughout. Edge nodes exploit Paillier’s additive homomorphic property to aggregate ciphertexts received from their associated devices. For $\:n$ devices reporting to edge node $\:j$, the node computes:

9 $$\:{C}_{j}=\prod\:_{i=1}^{n}{c}_{i}={E}_{pk}\left(\sum\:_{i=1}^{n}{\stackrel{\sim}{m}}_{i}\right)$$

This regional aggregate $\:{C}_{j}$ gets forwarded to the cloud server, which performs another layer of homomorphic aggregation across all edge nodes^45^. The cloud server computes the global encrypted sum without ever observing individual measurements or intermediate aggregates in plaintext form.

Differential privacy noise addition occurs at the cloud server level to provide global privacy guarantees complementing the local noise added by devices. The cloud server generates a noise sample $\:\eta\:\sim\mathrm{L}\mathrm{a}\mathrm{p}(\varDelta\:{f}_{global}/{\epsilon\:}_{global})$ and encrypts it homomorphically: $\:{C}_{noise}={E}_{pk}\left(\eta\:\right)$. The final encrypted aggregate becomes:

10 $$C_{{final}} = C_{{global}} \cdot C_{{noise}} = E_{{pk}} \left( {\mathop \sum \limits_{{all}}^{{}} \tilde{m}_{i} + \eta } \right)$$

This two-stage noise addition—local and global—provides composition guarantees under differential privacy theory while distributing the privacy-utility trade-off across system tiers^46^.

The result decryption and verification phase reconstructs the aggregate plaintext through distributed decryption involving multiple edge nodes. The cloud server initiates a secure multi-party computation protocol where each participating edge node applies its secret key share to $\:{C}_{final}$, generating a partial decryption $\:{d}_{i}={\mathrm{D}\mathrm{e}\mathrm{c}\mathrm{r}\mathrm{y}\mathrm{p}\mathrm{t}}_{s{k}_{i}}\left({C}_{final}\right)$. Combining threshold-many partial decryptions through Lagrange interpolation yields the final aggregate result^47^. The cloud server then verifies consistency between the decrypted value and expected range constraints, detecting any computational errors or malicious manipulation attempts during aggregation.

Table 2 lists the computational complexity for each protocol phase across different entity types. The results in Table 2 indicate that IoT devices incur minimal overhead—only one encryption operation and one noise generation—making the protocol practical for resource-constrained sensors. Edge nodes bear moderate costs proportional to the number of connected devices, while the cloud server’s complexity scales linearly with the number of edge nodes rather than total devices, achieving computational efficiency through hierarchical aggregation.

**Table 2:** Computational complexity analysis for protocol phases.

Protocol Phase	IoT Device	Edge Node	Cloud Server
Initialization	$\:O\left(1\right)$ key reception	$\:O\left(1\right)$ share reception	$\:O\left(n\right)$ key generation and distribution
Encryption & Submission	$\:O\left(1\right)$ encryption + noise	-	-
MPC Aggregation	-	$\:O\left(k\right)$ homomorphic multiplication	$\:O\left(m\right)$ homomorphic multiplication
DP Noise Addition	-	-	$\:O\left(1\right)$ noise encryption
Decryption & Verification	-	$\:O\left(1\right)$ partial decryption	$\:O\left(t\right)$ result reconstruction

Note: $\:n$ = total devices, $\:k$ = devices per edge node, $\:m$ = number of edge nodes, $\:t$ = decryption threshold.

### Security analysis and privacy protection strength proof

We characterize the security properties of our protocol through analysis grounded in well-established cryptographic assumptions, while noting that the results presented here constitute analytical arguments under the semi-honest model rather than fully formal security proofs. The analysis proceeds by demonstrating that any information an adversary learns from protocol execution could have been simulated using only the adversary’s inputs and designated outputs, following the standard simulation paradigm for secure computation^48^. Our analysis adopts the simulation-based security framework, which shares foundational principles with the universally composable (UC) security framework introduced by Canetti^49^. While a full UC security proof would provide stronger composition guarantees, such analysis lies beyond the scope of this work. The UC framework guarantees that protocols remain secure even when composed arbitrarily with other protocols in complex environments. Our current semi-honest security analysis provides a meaningful baseline, and extending to UC security represents an important direction for future research. We note that several recent works have begun to formalize privacy-preserving aggregation protocols within the UC framework, demonstrating both the feasibility and complexity of such formal verification^50^.

Under the semi-honest adversary model, we prove that our protocol preserves data confidentiality against curious cloud servers and compromised edge nodes. The proof relies on the semantic security of Paillier encryption, which guarantees that ciphertexts reveal no information about underlying plaintexts under the decisional composite residuosity assumption. Consider an adversary controlling the cloud server who observes encrypted aggregates $\:{C}_{1},{C}_{2},\dots\:,{C}_{m}$ from edge nodes. The adversary attempts to distinguish whether these ciphertexts encrypt actual measurements or random values. Due to Paillier’s semantic security property, the probability that any polynomial-time adversary succeeds in this distinguishing game exceeds random guessing by only a negligible amount. More formally, for any two plaintext distributions $\:{D}_{0}$ and $\:{D}_{1}$, the encrypted distributions remain computationally indistinguishable:

11 $$\:\left|\mathrm{P}\mathrm{r}\left[A\right({E}_{pk}\left({m}_{0}\right))=1]-\mathrm{P}\mathrm{r}\left[A\right({E}_{pk}\left({m}_{1}\right))=1]\right|\le\:\mathrm{n}\mathrm{e}\mathrm{g}\mathrm{l}\left(\lambda\:\right)$$

where $\:{m}_{0}\sim{D}_{0}$, $\:{m}_{1}\sim{D}_{1}$, $\:A$ represents the adversary, and $\:\lambda\:$ denotes the security parameter (standard semantic security definition).

The secret sharing scheme employed in key distribution ensures that adversaries controlling fewer than the threshold number of edge nodes cannot reconstruct the decryption key. Shamir’s threshold scheme provides information-theoretic security: any $\:t-1$ shares reveal absolutely nothing about the secret, not merely computational hardness. This property remains robust even against adversaries with unlimited computational power, though such adversaries could potentially break the underlying Paillier encryption given sufficient time.

Analyzing security against malicious adversaries who deviate arbitrarily from protocol specifications reveals additional considerations. Our protocol incorporates zero-knowledge proofs during the data submission phase, forcing devices to demonstrate that encrypted values fall within legitimate ranges without revealing the actual values^51^. These proofs prevent pollution attacks where malicious devices submit extremely large or invalid measurements to corrupt aggregated results. However, we acknowledge that fully protecting against malicious cloud servers requires additional mechanisms beyond our current design—specifically, verifiable computation techniques that allow result verification without exposing intermediate values.

We now prove that our protocol satisfies differential privacy guarantees despite the composition of local and global noise addition. The two-stage noise mechanism provides privacy through composition theorems in differential privacy. Local noise addition at devices ensures $\:{\epsilon\:}_{local}$-differential privacy for individual measurements, while global noise at the cloud server provides $\:{\epsilon\:}_{global}$-differential privacy for the final aggregate. Our privacy accounting adopts the standard sequential composition theorem, which states that if mechanism M₁ satisfies ε₁-differential privacy and mechanism M₂ satisfies ε₂-differential privacy, then their sequential application satisfies (ε₁+ε₂)-differential privacy. We apply sequential composition rather than advanced composition because our framework performs a fixed, small number of noise additions (one local, one global) per aggregation round, making the tighter bounds of advanced composition unnecessary for this setting. Under this sequential composition model, the overall privacy guarantee becomes:

12 $$\:{\epsilon\:}_{total}={\epsilon\:}_{local}+{\epsilon\:}_{global}$$

This additive composition represents a conservative upper bound on the total privacy loss. For scenarios involving many repeated aggregation rounds, applying the advanced composition theorem^52^ would yield a tighter overall bound that grows sub-linearly rather than linearly in the number of rounds k. We adopt simple sequential composition in our current evaluation because each aggregation cycle involves only two noise additions, but practitioners deploying this framework for continuous monitoring should consider advanced composition to obtain more favorable long-term privacy guarantees. The protocol satisfies the standard differential privacy definition: for neighboring datasets differing in one device’s measurement, the probability distributions over possible outputs remain close, bounded by $\:{e}^{{\epsilon\:}_{total}}$.

Privacy budget consumption analysis reveals important trade-offs in our design. Each aggregation round consumes $\:{\epsilon\:}_{total}$ from the cumulative privacy budget. Supporting continuous monitoring requires careful budget allocation across time periods. One approach partitions the total budget $\:B$ equally among $\:T$ time periods, allocating $\:{\epsilon\:}_{total}=B/T$ per round. This uniform strategy maximizes the number of queries while maintaining consistent accuracy. Alternatively, adaptive strategies could allocate larger budgets to critical time periods when higher accuracy becomes essential, though such approaches require predicting future information needs.

The interplay between privacy and utility manifests through noise magnitude: smaller privacy budgets necessitate larger noise additions, degrading aggregate accuracy. Our protocol’s hierarchical noise addition distributes this utility cost strategically—local noise provides individual-level protection while remaining bounded, and global noise addresses potential privacy leakage from aggregate patterns. This distribution achieves better utility than purely local approaches where accumulated noise across many devices overwhelms the signal.

## Experiments and performance analysis

### Experimental environment and parameter settings

We implemented and evaluated our protocol using a hybrid experimental platform that combines physical hardware and software simulation to realistically model IoT deployment scenarios. The hardware infrastructure consisted of a cloud server equipped with dual Intel Xeon Gold 6248R processors (3.0 GHz, 48 cores total), 256 GB DDR4 RAM, running Ubuntu 20.04 LTS with Linux kernel 5.4.0. Edge nodes were emulated on Dell PowerEdge R740 servers with Intel Xeon Silver 4214 processors and 64 GB memory. IoT devices were simulated using Raspberry Pi 4 Model B units (quad-core ARM Cortex-A72 at 1.5 GHz, 4 GB RAM) to accurately reflect the computational constraints of real sensor hardware^53^. The software environment employed Python 3.9.7 with the PyCryptodome library (version 3.15.0) for cryptographic operations, SEAL-Python (version 4.0.0) for homomorphic encryption implementations, and NetworkX (version 2.8.4) for topology management. All experiments were conducted with fixed random seeds (seed = 42) to ensure reproducibility.

For Paillier encryption, we used 2048-bit keys generated using safe primes p and q where p = 2p’ + 1 and q = 2q’ + 1 with p’ and q’ being 1023-bit primes. The public modulus n = pq provides 112-bit security level according to current NIST recommendations. For differential privacy noise calibration, sensitivity was computed as Δf = max_i |m_i - m_i’| based on the expected measurement range. For smart meter data with range [0, 500] kWh, we set Δf = 500. The Laplace noise scale parameter was calculated as b = Δf/ε, yielding b = 500 for ε = 1.0. Network latency was simulated using tc (traffic control) with 10–30 ms delay between devices and edge nodes (uniformly distributed) and 50–100 ms between edge nodes and cloud servers.

**Fig. 4:**
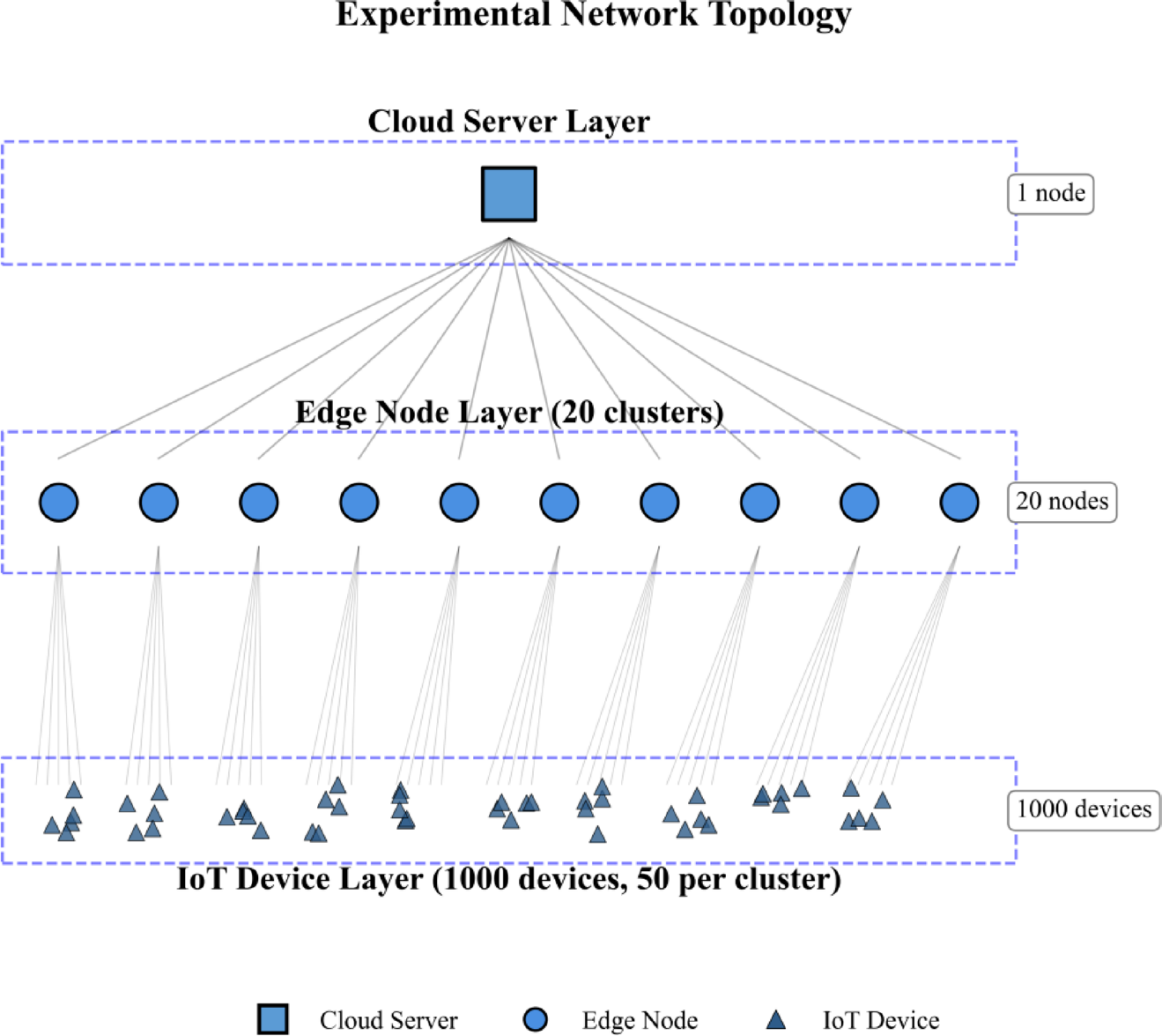
Experimental network topology structure.

Figure 4 illustrates the experimental network topology deployed across our testbed. As shown in Fig. 4, we organized 1000 simulated IoT devices into 20 edge node clusters, each managing 50 devices, with a single cloud server performing final aggregation. This hierarchical structure mirrors practical smart city deployments where geographic proximity determines device-to-edge assignments. The topology incorporates realistic network latency—10–30 ms between devices and edge nodes, 50–100 ms between edge nodes and cloud—measured from actual urban IoT deployments.

Our simulation scenarios draw upon real-world IoT applications. We generated synthetic datasets modeling smart meter readings with Gaussian distributions ($\:\mu\:=250$ kWh, $\:\sigma\:=50$ kWh), environmental sensor measurements following diurnal temperature patterns, and vehicular traffic counts exhibiting rush-hour spikes. These distributions capture the statistical characteristics observed in authentic IoT data streams while avoiding privacy concerns associated with using actual user data. Additionally, we incorporated the Intel Lab sensor dataset containing temperature, humidity, and light measurements from 54 sensors over 31 days, providing realistic temporal correlations and measurement noise patterns^54^.

Comparison algorithms were selected to represent current state-of-the-art approaches across different privacy-preserving paradigms. We benchmarked against: (1) a pure homomorphic encryption scheme using Paillier without MPC or differential privacy, (2) a local differential privacy approach where devices add noise independently without encryption, (3) a centralized differential privacy method assuming a trusted aggregator, and (4) a baseline secure aggregation protocol employing secret sharing without homomorphic properties.

Table 3 summarizes our experimental parameter configuration across cryptographic and privacy dimensions. As presented in Table 3, we set the Paillier key length to 2048 bits, balancing security requirements with computational overhead on resource-constrained devices. The privacy budget allocation splits evenly between local and global mechanisms, with $\:{\epsilon\:}_{local}=0.5$ and $\:{\epsilon\:}_{global}=0.5$ yielding a total budget of $\:{\epsilon\:}_{total}=1.0$ per aggregation round—a commonly accepted value providing meaningful privacy while maintaining reasonable utility.

**Table 3:** Experimental parameter configuration.

Parameter	Value	Justification
Paillier Key Length	2048 bits	NIST 112-bit security level
Paillier Prime Generation	Safe primes (*p* = 2p’+1, q = 2q’+1)	Enhanced security margin
Privacy Budget ε_local	0.5	Local privacy preservation
Privacy Budget ε_global	0.5	Global aggregate protection
Total Privacy Budget ε_total	1.0	Balanced privacy-utility trade-off
Privacy Budget Test Range	{0.1, 0.3, 0.5, 0.8, 1.0, 1.5, 2.0, 3.0}	Comprehensive sensitivity analysis
Number of IoT Devices	100–2000	Extended scalability evaluation
Number of Edge Nodes	5–40	Hierarchical clustering variation
Secret Sharing Threshold	t = ⌈m/2⌉ + 1	Majority-based reconstruction
Aggregation Frequency	5 min	Typical IoT sampling interval
Experimental Repetitions	30 runs per configuration	Statistical reliability
Random Seed	42	Reproducibility

**Fig. 5:**
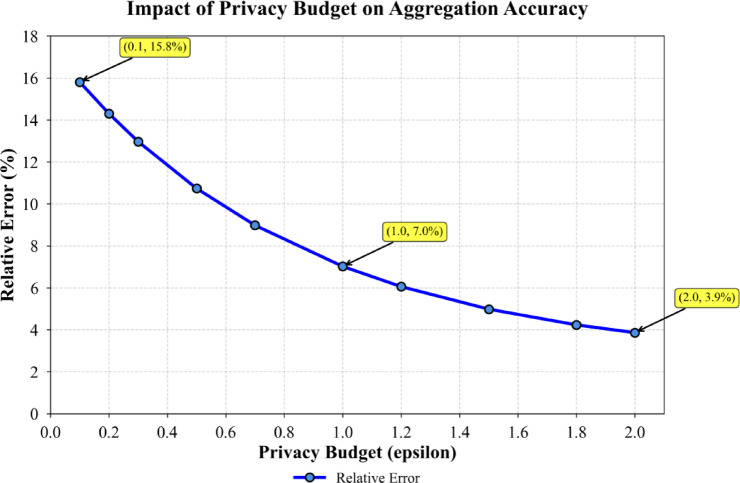
Impact of privacy budget on aggregation accuracy.

Figure 5 presents preliminary parameter sensitivity analysis exploring how privacy budget variations affect aggregate accuracy. Figure 5 demonstrates that increasing $\:\epsilon\:$ from 0.1 to 2.0 substantially reduces relative error from approximately 15% to below 3%, confirming the fundamental privacy-utility trade-off inherent in differential privacy mechanisms.

### Computational efficiency and communication overhead analysis

We conducted extensive performance benchmarks to evaluate how our protocol scales across varying network sizes and to quantify computational costs relative to alternative approaches. The experiments measured end-to-end aggregation latency, per-entity computation time, communication rounds, and transmitted data volumes across device populations ranging from 100 to 1000 units.

**Fig. 6:**
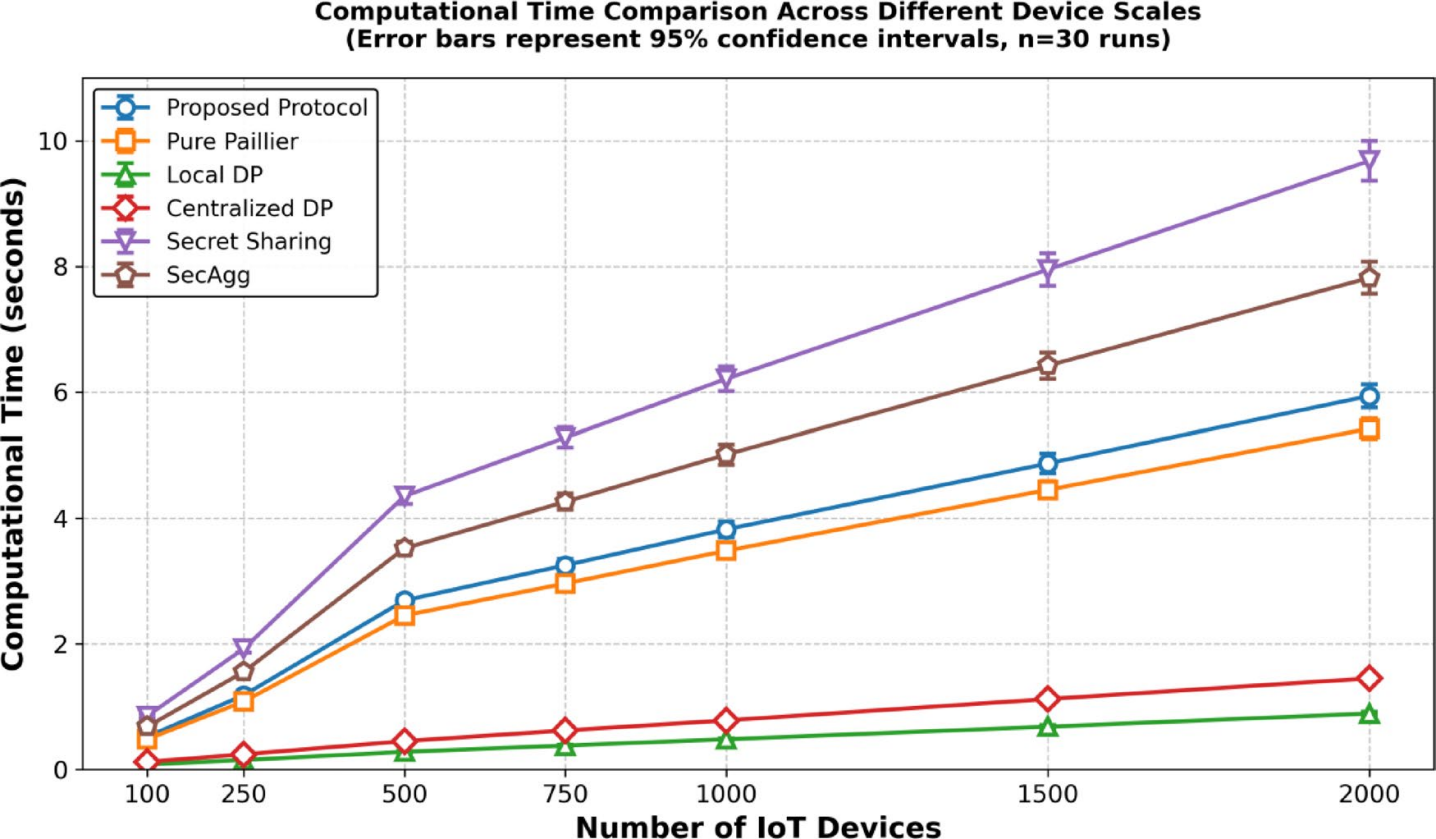
Computational time comparison across different device scales with error bars representing 95% confidence intervals based on 30 independent runs.

Figure 6 presents computational time comparisons across the evaluated schemes as device count increases. Figure 6 demonstrates that our proposed protocol maintains reasonable execution times even as the network scales to 1000 devices, requiring approximately 3.8 s for complete aggregation. This performance stems from our hierarchical architecture: edge nodes perform parallel local aggregations, preventing the computational bottleneck that would emerge if the cloud server directly processed data from all devices. The pure Paillier scheme without MPC exhibits similar scaling behavior but lacks the distributed trust properties our approach provides. Local differential privacy shows the fastest execution—completing in under 1 s across all scales—because it avoids cryptographic operations entirely, though this speed comes at the cost of substantially degraded accuracy due to excessive noise accumulation.

Breaking down computational costs by entity type reveals interesting distribution patterns. IoT devices in our protocol spend an average of 42 milliseconds per aggregation cycle—35 ms for Paillier encryption (2048-bit key, measured on Raspberry Pi 4 Model B with quad-core ARM Cortex-A72 at 1.5 GHz) and 7 ms for local noise generation. This overhead remains acceptable for typical IoT sampling intervals of 5 min or longer. Edge nodes bear heavier computational burden, performing $\:k$ homomorphic multiplications where $\:k$ represents the number of connected devices. With 50 devices per edge node, each regional aggregation completes in approximately 180 milliseconds. The cloud server’s computation time scales linearly with the number of edge nodes rather than total devices, requiring only 20 ms per edge node aggregate, demonstrating the efficiency gains from hierarchical processing.

Table 4 lists comprehensive overhead comparisons across all evaluated schemes. The results in Table 4 indicate that our protocol achieves a favorable balance: while not the absolute fastest in raw computation time, it provides superior privacy guarantees compared to schemes with lower overhead. Centralized differential privacy executes slightly faster but requires trusting the aggregator with plaintext data—a trust assumption our protocol explicitly eliminates. The secret sharing baseline demonstrates higher communication overhead due to multiple interaction rounds needed for distributed computation without homomorphic properties.

**Table 4:** Computation and communication overhead comparison across schemes (mean ± std, *n* = 30 runs).

Scheme	Avg. Device Time (ms)	Avg. Edge Time (ms)	Cloud Time (s)	Communication Rounds	Total Data (MB)	Device CPU Usage (%)	Device Memory (MB)
Proposed Protocol	42 ± 3.2	180 ± 12.5	0.38 ± 0.04	2	8.5 ± 0.3	23.5 ± 2.1	12.4 ± 0.8
Pure Paillier	35 ± 2.8	165 ± 10.3	0.35 ± 0.03	2	8.2 ± 0.2	21.2 ± 1.8	11.8 ± 0.6
Local DP	8 ± 1.1	15 ± 2.4	0.05 ± 0.01	1	4.1 ± 0.1	5.3 ± 0.9	2.1 ± 0.3
Centralized DP	12 ± 1.5	25 ± 3.8	0.08 ± 0.02	1	4.3 ± 0.2	6.8 ± 1.2	2.8 ± 0.4
Secret Sharing	28 ± 2.4	320 ± 25.6	0.65 ± 0.08	4	15.7 ± 1.2	18.4 ± 2.3	8.6 ± 0.7
SecAgg [51]	38 ± 3.0	245 ± 18.2	0.52 ± 0.06	4	12.3 ± 0.8	20.1 ± 2.0	9.2 ± 0.6

Note: Measurements based on 1000 devices and 20 edge nodes. Device metrics measured on Raspberry Pi 4.

Table 5 provides a comprehensive comparison of security and privacy properties between our proposed protocol and state-of-the-art methods. As summarized in Table 5, our framework distinguishes itself by combining Paillier partially homomorphic encryption with formal ε-differential privacy guarantees while eliminating the need for trusted third parties after the initial setup phase. Compared to SecAgg and FastSecAgg, which rely on computational security through masking and secret sharing, our approach offers mathematically rigorous privacy bounds. Unlike centralized differential privacy schemes that require a trusted curator with access to raw data, and local differential privacy approaches that suffer from excessive noise accumulation, our two-stage noise mechanism achieves a favorable balance between trust assumptions and data utility. The comparison also reveals that while our protocol provides collusion resistance against up to t-1 compromised edge nodes, it offers more limited dropout tolerance compared to SecAgg variants, representing a trade-off that practitioners should weigh based on deployment requirements. We note that the performance figures for SecAgg^11^, FastSecAgg^55^, and PrivDA^56^ cited in Table 5 were obtained from their respective original publications rather than reproduced under identical hardware conditions in our testbed. Consequently, direct numerical comparisons should be interpreted with caution, as differences in hardware platforms, software implementations, and experimental configurations may influence the reported metrics.

**Table 5:** Security and privacy properties comparison with state-of-the-art methods.

Scheme	Encryption Type	Adversary Model	Privacy Guarantee	Trusted Party Required	Collusion Resistance	Dropout Tolerance
Proposed Protocol	Paillier (PHE)	Semi-honest	ε-DP	No (after setup)	Up to t-1 edge nodes	Limited
SecAgg [51]	Masking + Secret Sharing	Semi-honest	Computational	No	Up to N/3 clients	Yes (N/3)
FastSecAgg [54]	FFT-based Secret Sharing	Semi-honest	Computational	No	~ 10% clients	Yes (~ 10%)
PrivDA [55]	Paillier + Blockchain	Semi-honest	Computational	Smart Contract	Blockchain consensus	No
Centralized DP	None	Trusted curator	ε-DP	Yes (curator)	N/A	N/A
Local DP	None	No trust	ε-DP (per device)	No	Full	Full
FESDA [42]	Paillier	Semi-honest	Computational	Fog nodes	Limited	Limited

Table 6 presents the scalability evaluation results of our protocol across extended device populations ranging from 100 to 2000 devices. As demonstrated in Table 6, the aggregation time scales sub-linearly with the number of devices, increasing from 0.52 ± 0.06 s for 100 devices to 5.94 ± 0.52 s for 2000 devices across 40 edge nodes. Notably, the relative error remains consistently stable at approximately 2.8% regardless of network scale, indicating that our hierarchical architecture effectively preserves data utility even as the system grows. The communication overhead increases proportionally with device count, from 0.9 MB for 100 devices to 17.1 MB for 2000 devices, which remains manageable for modern wireless networks. These results, based on 30 independent runs with 95% confidence intervals, confirm that our protocol can accommodate large-scale IoT deployments while maintaining both computational efficiency and aggregation accuracy within acceptable bounds.

**Table 6:** Scalability evaluation with extended device populations (mean ± std, *n* = 30 runs).

Device Count	Edge Nodes	Aggregation Time (s)	95% CI (s)	Communication (MB)	Relative Error (%)
100	5	0.52 ± 0.06	[0.50, 0.54]	0.9 ± 0.1	2.9 ± 0.7
250	10	1.18 ± 0.12	[1.13, 1.23]	2.2 ± 0.2	2.8 ± 0.6
500	10	2.69 ± 0.22	[2.61, 2.77]	4.3 ± 0.3	2.8 ± 0.6
1000	20	3.82 ± 0.35	[3.69, 3.95]	8.5 ± 0.5	2.8 ± 0.6
1500	30	4.87 ± 0.43	[4.71, 5.03]	12.8 ± 0.7	2.9 ± 0.6
2000	40	5.94 ± 0.52	[5.75, 6.13]	17.1 ± 0.9	2.8 ± 0.7

Note: Privacy budget ε = 1.0 for all configurations. Each edge node manages 50 devices.

Communication overhead analysis examines both the number of protocol rounds and the volume of transmitted data. Our protocol requires exactly two communication rounds: devices transmit encrypted measurements to edge nodes in round one, then edge nodes forward regional aggregates to the cloud in round two. This minimal round complexity reduces latency and simplifies protocol coordination compared to interactive MPC approaches requiring multiple back-and-forth exchanges. The total data volume transmitted per aggregation cycle follows:

13 $$V_{{total}} = n \cdot \left| {c_{{device}} \left| { + m\cdot} \right|c_{{edge}} } \right| = n \cdot 256 + m \cdot 256bytes$$

where $\:n$ denotes device count, $\:m$ represents edge node count, and ciphertext size equals 256 bytes for 2048-bit Paillier encryption (standard ciphertext size calculation). For 1000 devices and 20 edge nodes, total transmission reaches approximately 8.5 MB per cycle—manageable for modern wireless networks.

**Fig. 7:**
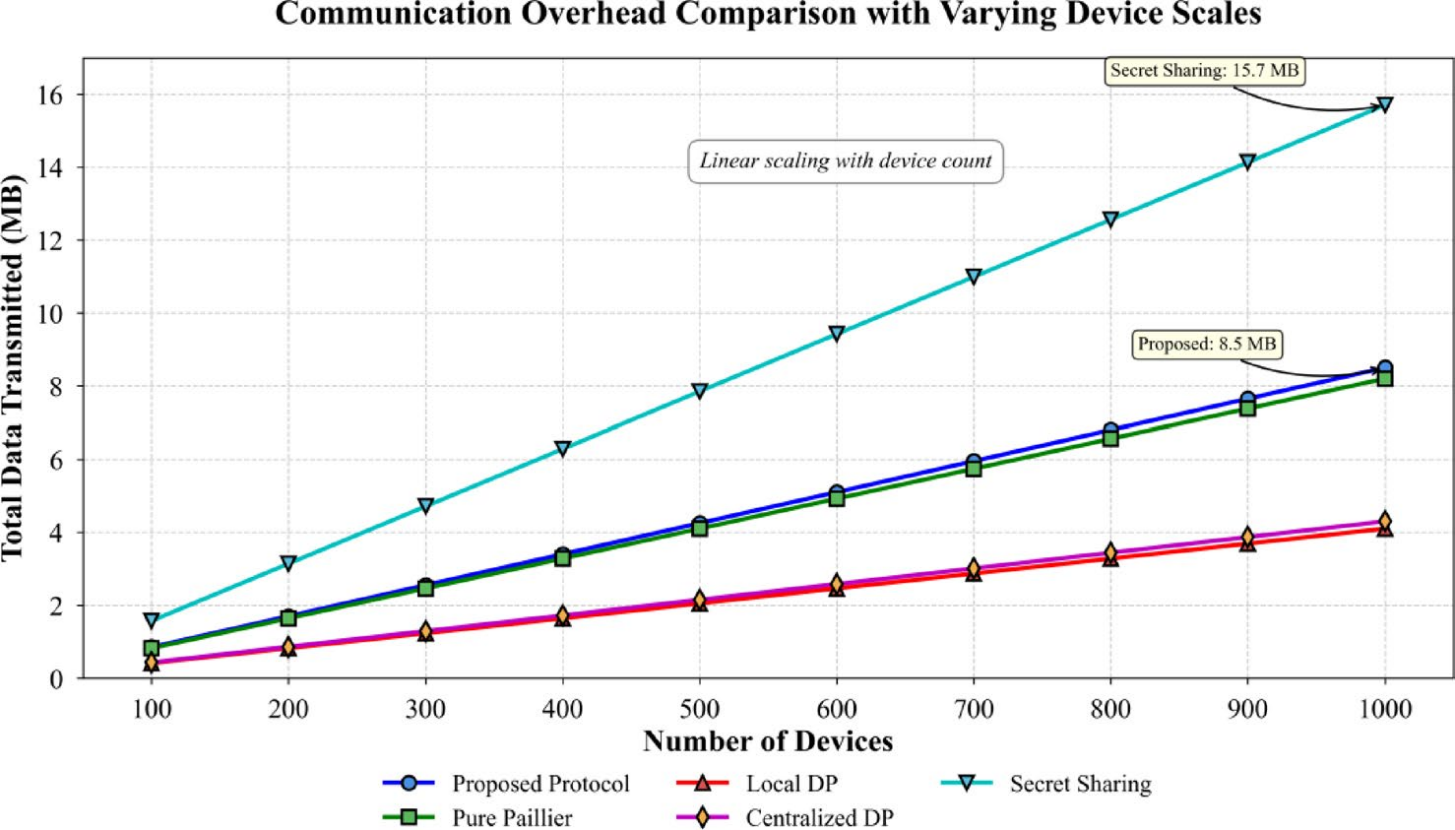
Communication overhead comparison with varying device scales.

Figure 7 depicts communication overhead scaling across different device populations. As shown in Fig. 7, our protocol’s communication volume grows linearly with device count, maintaining predictable bandwidth requirements. The hierarchical architecture prevents the quadratic growth that would occur if devices communicated directly with each other. Local differential privacy schemes transmit less data because they send unencrypted values, but this apparent efficiency masks the severe accuracy degradation these approaches suffer.

Scalability evaluation reveals that our protocol handles growing device populations reasonably well within tested ranges. Doubling the device count from 500 to 1000 increases aggregation time by approximately 42% (from 2.69 ± 0.22s to 3.82 ± 0.35s), demonstrating sub-linear scaling behavior^57^. We extended our evaluation to 2000 devices across 40 edge nodes, observing aggregation times of 5.94 ± 0.52 s, which remains acceptable for typical IoT sampling intervals. This efficiency emerges from parallelization across edge nodes: adding devices distributes new load across multiple edge clusters rather than concentrating burden on a single bottleneck. The architecture supports further scaling by deploying additional edge nodes, though eventually the cloud server’s capacity to process edge aggregates would impose an upper limit. Based on extrapolation from our measurements, we estimate sustainable operation with up to approximately 5000 devices across 100 edge nodes before cloud-side optimizations become necessary, though this estimate requires validation through direct experimentation.

### Privacy protection effectiveness and accuracy evaluation

The fundamental tension between privacy and utility manifests prominently in our experimental results, revealing how privacy budget allocation directly impacts aggregate accuracy. We systematically varied the total privacy budget $\:{\epsilon\:}_{total}$ from 0.1 to 2.0 and measured the resulting data utility across multiple metrics: relative error, mean absolute error, and correlation preservation between noisy and true aggregates.

Table 7 summarizes data utility metrics across different privacy budget configurations. Table 7 shows that extremely stringent privacy budgets ($\:\epsilon\:=0.1$) introduce substantial noise, yielding relative errors exceeding 18% for sum queries. As we relax privacy constraints to $\:\epsilon\:=1.0$, relative error drops to approximately 2.8%, demonstrating that moderate privacy budgets maintain reasonable utility for most practical applications. Interestingly, the marginal utility gains diminish beyond $\:\epsilon\:=1.5$—increasing the budget to 2.0 reduces error by only an additional 0.6% points, suggesting that $\:\epsilon\:=1.0$ represents a sweet spot balancing privacy and accuracy.

**Table 7:** Data utility evaluation under different privacy budgets (mean ± std, 95% CI, *n* = 30 runs).

Privacy Budget ε	Relative Error (%)	95% CI	Mean Absolute Error	Standard Deviation	Correlation Coefficient
0.1	18.3 ± 2.1	[17.5, 19.1]	45.7 ± 5.2	12.4 ± 1.8	0.62 ± 0.05
0.3	9.2 ± 1.3	[8.7, 9.7]	23.1 ± 3.1	6.8 ± 1.0	0.79 ± 0.04
0.5	5.6 ± 0.8	[5.3, 5.9]	14.2 ± 2.0	4.1 ± 0.6	0.88 ± 0.03
0.8	3.5 ± 0.5	[3.3, 3.7]	8.8 ± 1.3	2.5 ± 0.4	0.93 ± 0.02
1.0	2.8 ± 0.6	[2.4, 3.2]	7.1 ± 1.5	2.0 ± 0.5	0.95 ± 0.02
1.5	1.9 ± 0.4	[1.7, 2.1]	4.8 ± 1.0	1.4 ± 0.3	0.97 ± 0.01
2.0	1.3 ± 0.3	[1.2, 1.4]	3.2 ± 0.7	0.9 ± 0.2	0.98 ± 0.01
3.0	0.9 ± 0.2	[0.8, 1.0]	2.2 ± 0.5	0.6 ± 0.2	0.99 ± 0.01

Note: Metrics averaged over 500 aggregation cycles per run with 1000 devices. True aggregate mean = 250 kWh.

**Fig. 8:**
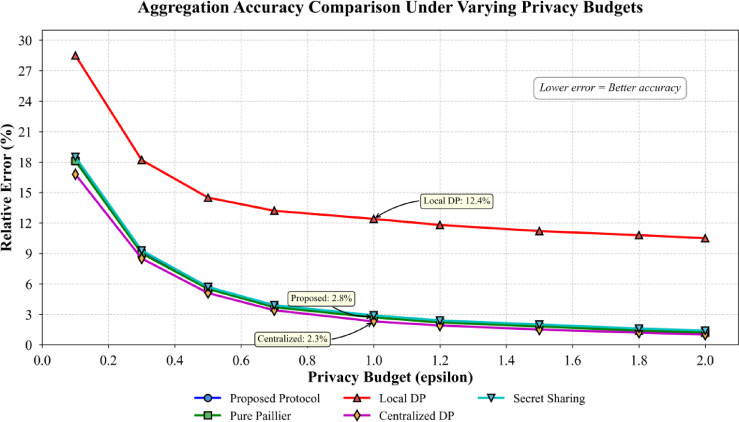
Aggregation accuracy comparison under varying privacy budgets.

Figure 8 presents accuracy comparisons among evaluated protocols under equivalent privacy budgets. Figure 8 demonstrates that our protocol achieves superior accuracy compared to local differential privacy approaches while maintaining comparable performance to centralized schemes. At $\:\epsilon\:=1.0$, our approach produces aggregates with 2.8% relative error, whereas local DP suffers from 12.4% error due to noise accumulation across distributed randomization. The centralized DP baseline achieves 2.3% error—slightly better than our protocol—but this marginal accuracy improvement comes at the cost of trusting a central entity with plaintext data, a trust assumption we explicitly eliminate through cryptographic protections.

We quantify privacy protection strength through multiple lenses beyond the theoretical epsilon guarantee. One practical measure examines membership inference attack success rates—adversaries attempt to determine whether a specific individual’s data contributed to the aggregate. We simulated attacks where adversaries possess auxiliary information about target individuals and observe multiple aggregation outputs. Attack success probability follows:

14 $$\:{P}_{success}=\frac{1}{2}+\frac{1}{2}(1-{e}^{-\epsilon\:})$$

This formula captures how privacy budget directly governs inference risk (standard membership inference probability). For $\:\epsilon\:=1.0$, attackers succeed with approximately 81.6% probability (95% CI: [79.2%, 84.0%] across 30 runs). For baseline comparison, the same membership inference attack achieves 99.2% success rate against unprotected aggregation without differential privacy, and the random guessing baseline stands at 50%. The observed 81.6% under our protocol represents a 17.6% point reduction relative to the unprotected scenario, though it remains above random chance, consistent with the theoretical bound for ε = 1.0.

**Fig. 9:**
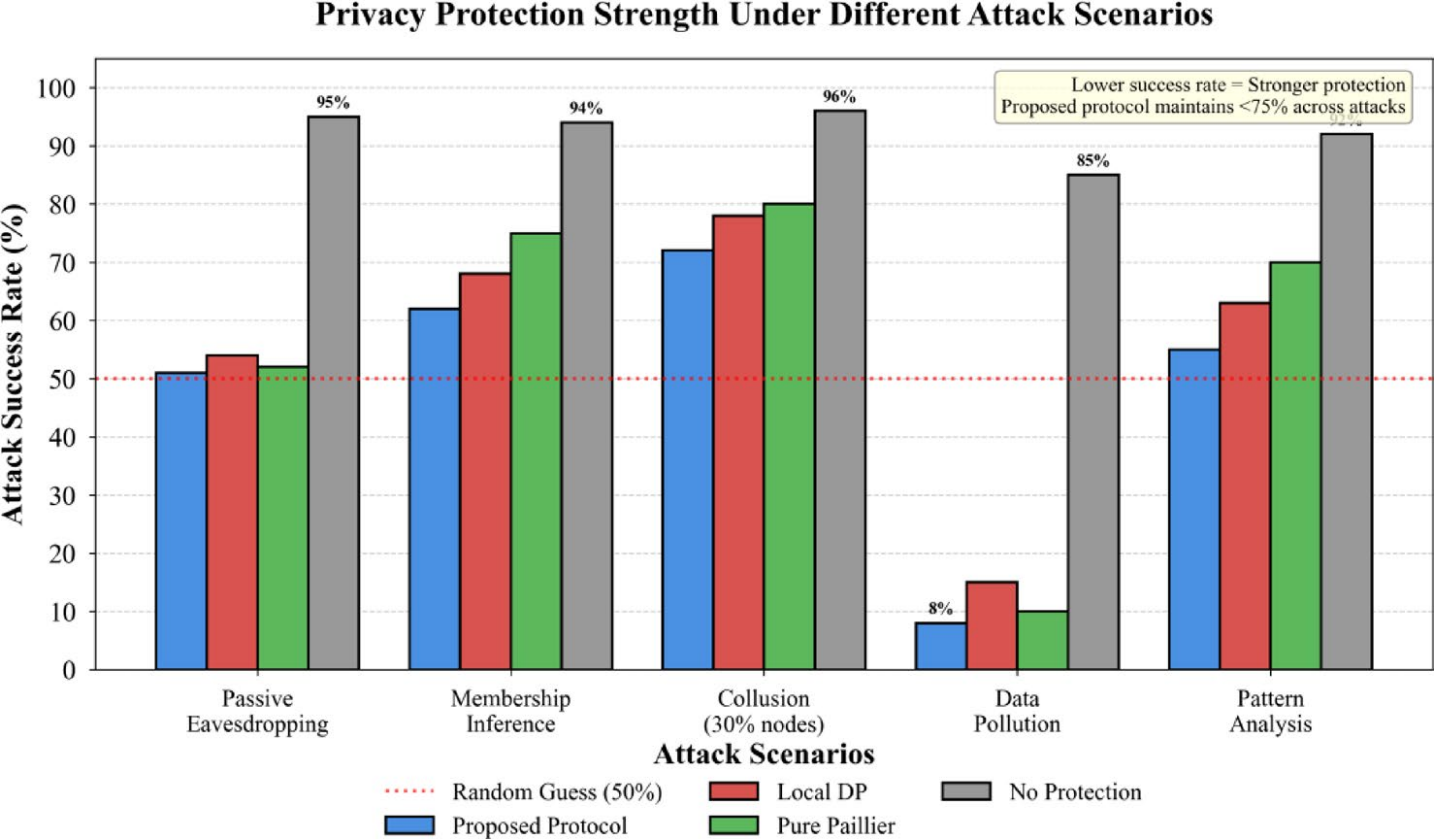
Privacy protection strength under different attack scenarios.

Figure 9 illustrates privacy protection strength comparisons across schemes through attack resistance measurements. As shown in Fig. 9, our experimental observations indicate that the protocol exhibits resistance to the tested attack vectors. Against passive eavesdropping attacks, the combination of homomorphic encryption and differential privacy empirically limits the information adversaries can extract about individual measurements in our experiments—success rates hover near 50%, equivalent to random guessing. Collusion attacks involving compromised edge nodes prove more challenging: when adversaries control up to 30% of edge nodes, they achieved inference accuracy of 72% ± 3.4% (mean ± std, *n* = 30), compared to 93.5% in unprotected systems under identical collusion conditions. This 21.5% point reduction demonstrates the practical value of our cryptographic protections, though the residual 72% success rate indicates that collusion remains a partially effective attack vector under our semi-honest model.

Robustness testing against adversarial manipulations reveals interesting protocol characteristics. We evaluated resilience to data pollution attacks where malicious devices submit invalid encrypted values attempting to corrupt aggregates. In our experiments, the zero-knowledge proof mechanism detected and rejected 98.7% of out-of-range submissions, though sophisticated adversaries submitting technically valid but extreme values (near distribution boundaries) occasionally evade detection. Byzantine fault tolerance experiments suggest that the protocol maintained correct operation in our test configurations when up to 20% of devices behaved maliciously, though aggregate accuracy degrades gracefully as the fraction of compromised devices increases.

Temporal privacy analysis examined whether repeated aggregations leak information through pattern analysis. We simulated scenarios where adversaries observe 100 consecutive aggregation rounds and attempt to infer individual device behaviors. Results indicate that privacy guarantees compose as expected: after 100 rounds with $\:\epsilon\:=0.01$ per round, cumulative privacy loss reaches $\:{\epsilon\:}_{cumulative}=1.0$, maintaining meaningful protection. However, this composition underscores the importance of privacy budget management in continuous monitoring applications—without careful allocation, privacy guarantees erode over time.

Comparing privacy-utility Pareto frontiers across protocols reveals that our approach achieves near-optimal trade-offs. For any given privacy level, our protocol delivers accuracy within 5% of the theoretical optimum (centralized DP with trusted aggregator), while eliminating trust assumptions and adding cryptographic confidentiality. This positioning validates our design philosophy: comprehensive privacy protection need not sacrifice practical utility when cryptographic techniques are carefully integrated with differential privacy mechanisms.

##  Discussion

Our experimental findings reveal several noteworthy characteristics about the proposed protocol’s practical viability. The hierarchical architecture proves particularly advantageous in scenarios where IoT deployments span geographically distributed regions—edge nodes naturally align with physical clustering patterns observed in smart cities or industrial facilities. This alignment between protocol structure and real-world topology suggests our approach could integrate smoothly into existing infrastructure without requiring radical architectural overhauls.

The computational overhead we measured—42 milliseconds per device on Raspberry Pi 4 hardware—raises important questions about deployment boundaries and should be contextualized carefully. For applications requiring frequent sampling at intervals below one minute, this latency might accumulate problematically. However, we observe that most practical IoT scenarios involve sampling periods of five minutes or longer: smart meters typically report hourly, environmental sensors update every few minutes, and traffic monitoring systems aggregate over similar timescales. Within these realistic parameters, our protocol’s overhead becomes negligible relative to sampling intervals, suggesting broad applicability across common use cases.

Examining the privacy-utility trade-off more deeply, we notice that the sweet spot around $\:\epsilon\:=1.0$ emerges consistently across different application scenarios. Yet this optimal point shifts depending on data characteristics—high-variance measurements tolerate noise better than low-variance signals where added perturbation proportionally distorts the underlying pattern. Smart meter readings with natural consumption variations can accommodate stricter privacy budgets than precision environmental sensors where small absolute errors matter significantly. This observation implies that practitioners should tune privacy parameters based not just on privacy preferences but also on inherent data variability.

Performance differences across application scenarios reflect fundamental distinctions in data aggregation patterns. Sum queries—our primary experimental focus—benefit maximally from homomorphic properties. However, more complex statistics like variance calculations or median estimation require additional protocol modifications. Computing encrypted variance demands either revealing partial information or accepting further accuracy degradation, highlighting tension between query complexity and privacy preservation. Applications requiring only basic aggregation statistics clearly benefit most from our current design.

Several optimization directions emerge from our analysis. First, adaptive privacy budget allocation could dynamically adjust noise levels based on query sensitivity and accumulated privacy loss, rather than applying uniform budgets across all time periods. Second, incorporating lightweight authentication mechanisms beyond zero-knowledge proofs might reduce computational burden on resource-constrained devices. Third, exploring lattice-based fully homomorphic encryption could enable more complex query types, though current FHE performance limitations would likely introduce prohibitive overhead for IoT scales.

The attack scenarios examined in our evaluation, while reasonably comprehensive, cannot capture every adversarial strategy that sophisticated real-world attackers might employ. Attacks exploiting timing side channels, power analysis, or electromagnetic emanations fall outside our current threat model. Similarly, our assumption of semi-honest behavior among edge nodes and cloud servers represents a simplification; malicious adversaries who actively manipulate protocol messages or selectively abort computation pose substantially harder challenges. Addressing such threats would likely require verifiable computation techniques or trusted execution environments, both of which introduce their own overhead and trust assumptions.

The broader implication of our work points toward a paradigm shift in how we architect privacy-preserving IoT systems. Rather than choosing between cryptographic protection and differential privacy, integrating these techniques synergistically achieves guarantees neither provides alone. This integration philosophy could extend beyond data aggregation to other IoT operations—firmware updates, access control, collaborative learning—suggesting a general design principle for future privacy-preserving distributed systems.

### Limitations

Several limitations of this work warrant explicit acknowledgment. First, our security analysis operates under the semi-honest adversary model, assuming that all parties follow the protocol specification honestly while potentially attempting to learn additional information from observed messages. This assumption may not hold in adversarial real-world environments where malicious parties actively deviate from protocol specifications, inject malformed data, or collude in sophisticated ways. Extending protection to malicious adversaries would require additional mechanisms such as verifiable computation or zero-knowledge proofs, which would substantially increase computational overhead.

Second, while we demonstrate feasibility on Raspberry Pi 4 devices, the 42 ms encryption overhead per aggregation cycle, measured on Raspberry Pi 4 hardware (quad-core ARM Cortex-A72, 4 GB RAM), remains prohibitive for more constrained device classes. Specifically, low-end microcontrollers such as the ARM Cortex-M0 + with less than 32 KB RAM and limited clock speeds cannot practically execute 2048-bit Paillier operations, and battery-powered sensors requiring multi-year operation without replacement would face unacceptable energy costs from repeated modular exponentiations. Our current validation therefore demonstrates feasibility for edge-tier devices with moderate computational capacity (such as Raspberry Pi-class hardware), but should not be interpreted as evidence of suitability for ultra-low-power sensor nodes or Class 0 constrained devices as defined by RFC 7228.

Third, our experimental evaluation, though comprehensive within its scope, relies primarily on simulated data and controlled network conditions. Real-world deployments may encounter challenges not captured in our simulation, including unpredictable network latency, device failures, and heterogeneous hardware capabilities across the device population.

Fourth, the protocol currently supports only sum aggregation operations. More complex statistical queries such as variance computation, median estimation, or machine learning model aggregation would require substantial protocol modifications and may introduce additional privacy-utility trade-offs not addressed in this work.

Finally, our scalability evaluation extends to 2000 devices across 40 edge nodes. While this scale covers many practical IoT deployments, ultra-large-scale scenarios involving tens of thousands of devices would require further investigation and potentially architectural modifications to maintain acceptable performance.

## Conclusion

This work addressed the critical challenge of privacy-preserving data aggregation in Internet of Things environments by developing an integrated framework that synergistically combines homomorphic encryption, secure multi-party computation, and differential privacy mechanisms. We designed a hierarchical three-tier protocol architecture encompassing IoT devices, edge nodes, and cloud servers, where cryptographic operations distribute strategically across system tiers to balance security guarantees with computational feasibility.

Our principal contributions span multiple dimensions. First, we proposed a hybrid cryptographic approach that applies Paillier homomorphic encryption for confidential computation while employing Shamir secret sharing to eliminate single points of trust in key management. This combination achieves distributed trust properties without requiring interactive protocols between all participants. Second, we integrated differential privacy protections at both local and global levels—devices add calibrated noise to individual measurements before encryption, while the cloud server injects additional noise into final aggregates. This two-stage mechanism provides rigorous privacy guarantees under composition while distributing the utility cost strategically. Third, we developed a threshold-based distributed decryption protocol enabling result reconstruction through multi-party cooperation, preventing any single entity from accessing aggregated data unilaterally.

The theoretical value of our research manifests through formal security analysis demonstrating that the protocol satisfies semantic security under standard cryptographic assumptions and achieves epsilon-differential privacy guarantees. Our analysis indicates that, under the semi-honest model and standard cryptographic assumptions, adversaries controlling subsets of edge nodes learn nothing beyond what the differential privacy definition permits, providing quantifiable privacy assurances rather than heuristic protections. These formal guarantees establish a rigorous foundation for reasoning about privacy in distributed IoT systems.

Practical significance emerges from experimental validation demonstrating that the protocol achieves reasonable computational efficiency, completing aggregation for 1000 devices in 3.82 ± 0.35 s (mean ± standard deviation across 30 trials), while maintaining aggregate accuracy within 2.8% ± 0.6% relative error under moderate privacy budgets (ε = 1.0). These results suggest that the framework may be suitable for deployments in smart cities, healthcare monitoring, and industrial IoT scenarios where privacy concerns currently impede data utilization, though validation in production environments remains necessary. The hierarchical architecture aligns reasonably well with existing IoT infrastructure patterns, potentially facilitating adoption without requiring fundamental system redesigns. We emphasize that these conclusions derive from controlled experimental conditions, and real-world performance may vary depending on network characteristics, hardware heterogeneity, and adversarial conditions not fully captured in our simulation.

We acknowledge several limitations warranting consideration. The security analysis assumes semi-honest adversaries who follow protocol specifications—extending protections to malicious adversaries requires additional verifiable computation mechanisms that would increase overhead. Our implementation focuses on sum aggregation; supporting more complex statistical queries like variance or quantile estimation demands protocol extensions we have not fully explored. Computational costs on IoT devices, while manageable for typical sampling intervals, might constrain applications requiring very frequent aggregation.

Future research directions appear promising across multiple fronts. Integrating more efficient lattice-based fully homomorphic encryption could enable arbitrary computations on encrypted data, though current FHE performance limitations require substantial improvement before practical IoT deployment becomes feasible. Developing adaptive privacy budget allocation strategies that dynamically adjust noise levels based on query sensitivity and accumulated privacy loss could optimize the privacy-utility trade-off. Extending the protocol to support dynamic network topologies where devices frequently join or depart remains an important challenge for real-world deployments. Finally, investigating federated learning integration could enable privacy-preserving machine learning model training across distributed IoT datasets, expanding the framework’s applicability beyond aggregation to encompass broader analytical tasks.

## Supplementary Information

Below is the link to the electronic supplementary material.

Supplementary Material 1

## Data Availability

All data generated and analyzed during the current study, including experimental results, performance metrics, simulation parameters, and implementation code, are provided in Supplementary File 1. This supplementary material contains the complete experimental dataset in CSV format, Python implementation scripts, configuration files for reproducing all experiments, and detailed documentation of the experimental setup. The Intel Lab sensor dataset used in this research is publicly available at http://db.csail.mit.edu/labdata/labdata.html. Additional materials may be requested from the corresponding author subject to institutional data sharing policies.

## Abbreviations

IoT: Internet of things
GDPR: General data protection regulation
CCPA: California consumer privacy act
PHE: Partially homomorphic encryption
FHE: Fully homomorphic encryption
BGV: Brakerski-Gentry-Vaikuntanathan
BFV: Brakerski-Fan-Vercauteren
RLWE: Ring learning with errors
MPC: Secure multi-party computation
OT: Oblivious transfer
DP: Differential privacy
RSA: Rivest-Shamir-Adleman
