# Supplementary material for: An integrated privacy preserving data aggregation framework for IoT networks using homomorphic encryption and secure computation
Source: Sci Rep. 2026 Apr 28;16:19663. doi: 10.1038/s41598-026-48831-6 (PMC13316016; doi:10.1038/s41598-026-48831-6)
Supplement: Supplementary file 1 — Supplementary Material 1 [file 41598_2026_48831_MOESM1_ESM.docx]

**Supplementary File 1: Experimental Data and Implementation Code**

**Supplementary Table S1: Raw Computational Efficiency Data (30 Trials, 1000 Devices, 20 Edge Nodes)**

| **Trial** | **Device Encryption Time (ms)** | **Edge Aggregation Time (ms)** | **Cloud Processing Time (ms)** | **Total Aggregation Time (s)** | **Communication Volume (MB)** |
| --- | --- | --- | --- | --- | --- |
| 1 | 43.2 | 178.5 | 382.1 | 3.78 | 8.42 |
| 2 | 41.8 | 182.3 | 391.2 | 3.85 | 8.51 |
| 3 | 44.1 | 176.9 | 378.5 | 3.71 | 8.38 |
| 4 | 40.5 | 185.2 | 395.8 | 3.92 | 8.55 |
| 5 | 42.7 | 179.8 | 384.3 | 3.81 | 8.47 |
| 6 | 43.9 | 181.1 | 387.6 | 3.86 | 8.49 |
| 7 | 41.2 | 177.4 | 376.9 | 3.69 | 8.41 |
| 8 | 44.5 | 183.7 | 392.4 | 3.91 | 8.53 |
| 9 | 42.3 | 180.2 | 385.1 | 3.82 | 8.48 |
| 10 | 40.9 | 178.9 | 381.7 | 3.77 | 8.44 |
| 11 | 43.6 | 184.5 | 394.2 | 3.93 | 8.54 |
| 12 | 41.5 | 176.2 | 375.8 | 3.68 | 8.39 |
| 13 | 44.2 | 181.8 | 388.9 | 3.87 | 8.50 |
| 14 | 42.1 | 179.5 | 383.6 | 3.80 | 8.46 |
| 15 | 40.7 | 182.9 | 390.5 | 3.89 | 8.52 |
| 16 | 43.4 | 177.1 | 377.2 | 3.70 | 8.40 |
| 17 | 41.9 | 180.6 | 386.4 | 3.84 | 8.49 |
| 18 | 44.8 | 183.2 | 393.1 | 3.92 | 8.54 |
| 19 | 42.5 | 178.3 | 380.9 | 3.76 | 8.43 |
| 20 | 40.3 | 181.5 | 389.2 | 3.88 | 8.51 |
| 21 | 43.1 | 179.1 | 382.8 | 3.79 | 8.45 |
| 22 | 41.7 | 184.8 | 396.5 | 3.95 | 8.56 |
| 23 | 44.4 | 176.5 | 374.6 | 3.66 | 8.37 |
| 24 | 42.8 | 180.9 | 387.3 | 3.85 | 8.50 |
| 25 | 40.6 | 178.7 | 381.4 | 3.77 | 8.44 |
| 26 | 43.7 | 182.6 | 391.8 | 3.90 | 8.53 |
| 27 | 41.4 | 177.8 | 378.2 | 3.72 | 8.41 |
| 28 | 44.6 | 181.3 | 388.6 | 3.87 | 8.50 |
| 29 | 42.2 | 183.9 | 394.9 | 3.94 | 8.55 |
| 30 | 40.8 | 179.4 | 383.2 | 3.80 | 8.46 |
| Mean | 42.37 | 180.32 | 385.97 | 3.82 | 8.48 |
| Std | 1.38 | 2.71 | 6.52 | 0.08 | 0.06 |
| 95% CI Lower | 41.85 | 179.30 | 383.53 | 3.79 | 8.46 |
| 95% CI Upper | 42.89 | 181.34 | 388.41 | 3.85 | 8.50 |

**Supplementary Table S2: Privacy Budget Sensitivity Analysis Raw Data (ε = 0.1 to 3.0)**

| **Trial** | **ε=0.1 RE(%)** | **ε=0.3 RE(%)** | **ε=0.5 RE(%)** | **ε=0.8 RE(%)** | **ε=1.0 RE(%)** | **ε=1.5 RE(%)** | **ε=2.0 RE(%)** | **ε=3.0 RE(%)** |
| --- | --- | --- | --- | --- | --- | --- | --- | --- |
| 1 | 17.8 | 9.1 | 5.4 | 3.4 | 2.7 | 1.8 | 1.2 | 0.8 |
| 2 | 18.9 | 9.5 | 5.8 | 3.6 | 2.9 | 2.0 | 1.4 | 0.9 |
| 3 | 16.5 | 8.7 | 5.2 | 3.2 | 2.5 | 1.7 | 1.1 | 0.7 |
| 4 | 19.2 | 9.8 | 6.1 | 3.8 | 3.1 | 2.1 | 1.5 | 1.0 |
| 5 | 17.4 | 8.9 | 5.3 | 3.3 | 2.6 | 1.7 | 1.2 | 0.8 |
| 6 | 18.6 | 9.4 | 5.7 | 3.5 | 2.8 | 1.9 | 1.3 | 0.9 |
| 7 | 16.8 | 8.5 | 5.1 | 3.1 | 2.4 | 1.6 | 1.0 | 0.7 |
| 8 | 19.5 | 9.7 | 5.9 | 3.7 | 3.0 | 2.0 | 1.4 | 1.0 |
| 9 | 17.2 | 9.0 | 5.5 | 3.4 | 2.7 | 1.8 | 1.2 | 0.8 |
| 10 | 18.1 | 9.2 | 5.6 | 3.5 | 2.8 | 1.9 | 1.3 | 0.9 |
| 11 | 19.8 | 9.9 | 6.2 | 3.9 | 3.2 | 2.2 | 1.6 | 1.1 |
| 12 | 16.2 | 8.4 | 4.9 | 3.0 | 2.3 | 1.5 | 0.9 | 0.6 |
| 13 | 18.4 | 9.3 | 5.7 | 3.6 | 2.9 | 1.9 | 1.3 | 0.9 |
| 14 | 17.6 | 8.8 | 5.4 | 3.3 | 2.6 | 1.7 | 1.1 | 0.8 |
| 15 | 19.1 | 9.6 | 5.8 | 3.7 | 3.0 | 2.0 | 1.4 | 1.0 |
| 16 | 16.9 | 8.6 | 5.2 | 3.2 | 2.5 | 1.6 | 1.0 | 0.7 |
| 17 | 18.3 | 9.1 | 5.5 | 3.4 | 2.7 | 1.8 | 1.2 | 0.8 |
| 18 | 19.4 | 9.8 | 6.0 | 3.8 | 3.1 | 2.1 | 1.5 | 1.0 |
| 19 | 17.1 | 8.7 | 5.3 | 3.3 | 2.6 | 1.7 | 1.1 | 0.8 |
| 20 | 18.7 | 9.4 | 5.7 | 3.5 | 2.8 | 1.9 | 1.3 | 0.9 |
| 21 | 16.6 | 8.5 | 5.1 | 3.1 | 2.4 | 1.6 | 1.0 | 0.7 |
| 22 | 19.7 | 9.9 | 6.1 | 3.9 | 3.2 | 2.2 | 1.5 | 1.1 |
| 23 | 17.5 | 8.9 | 5.4 | 3.4 | 2.7 | 1.8 | 1.2 | 0.8 |
| 24 | 18.2 | 9.2 | 5.6 | 3.5 | 2.8 | 1.9 | 1.3 | 0.9 |
| 25 | 16.4 | 8.4 | 5.0 | 3.0 | 2.3 | 1.5 | 0.9 | 0.6 |
| 26 | 19.3 | 9.7 | 5.9 | 3.7 | 3.0 | 2.0 | 1.4 | 1.0 |
| 27 | 17.3 | 8.8 | 5.3 | 3.3 | 2.6 | 1.7 | 1.1 | 0.8 |
| 28 | 18.5 | 9.3 | 5.6 | 3.5 | 2.8 | 1.9 | 1.3 | 0.9 |
| 29 | 19.6 | 9.8 | 6.0 | 3.8 | 3.1 | 2.1 | 1.5 | 1.0 |
| 30 | 17.0 | 8.6 | 5.2 | 3.2 | 2.5 | 1.6 | 1.0 | 0.7 |
| Mean | 18.03 | 9.13 | 5.55 | 3.47 | 2.74 | 1.85 | 1.23 | 0.85 |
| Std | 1.05 | 0.47 | 0.35 | 0.26 | 0.26 | 0.20 | 0.18 | 0.13 |

Note: RE = Relative Error (%)

**Supplementary Table S3: Scalability Evaluation Detailed Results**

| **Device Count** | **Edge Nodes** | **Trial** | **Aggregation Time (s)** | **Communication (MB)** | **Relative Error (%)** | **Device CPU (%)** | **Device Memory (MB)** |
| --- | --- | --- | --- | --- | --- | --- | --- |
| 100 | 5 | 1-10 avg | 0.51 | 0.88 | 2.85 | 23.2 | 12.1 |
| 100 | 5 | 11-20 avg | 0.53 | 0.91 | 2.92 | 23.6 | 12.5 |
| 100 | 5 | 21-30 avg | 0.52 | 0.89 | 2.88 | 23.4 | 12.3 |
| 250 | 10 | 1-10 avg | 1.15 | 2.18 | 2.78 | 23.3 | 12.2 |
| 250 | 10 | 11-20 avg | 1.21 | 2.24 | 2.82 | 23.7 | 12.6 |
| 250 | 10 | 21-30 avg | 1.18 | 2.21 | 2.80 | 23.5 | 12.4 |
| 500 | 10 | 1-10 avg | 2.65 | 4.25 | 2.76 | 23.4 | 12.3 |
| 500 | 10 | 11-20 avg | 2.73 | 4.35 | 2.81 | 23.8 | 12.7 |
| 500 | 10 | 21-30 avg | 2.69 | 4.30 | 2.79 | 23.6 | 12.5 |
| 1000 | 20 | 1-10 avg | 3.78 | 8.42 | 2.75 | 23.5 | 12.4 |
| 1000 | 20 | 11-20 avg | 3.86 | 8.54 | 2.82 | 23.9 | 12.8 |
| 1000 | 20 | 21-30 avg | 3.82 | 8.48 | 2.79 | 23.7 | 12.6 |
| 1500 | 30 | 1-10 avg | 4.82 | 12.65 | 2.86 | 23.6 | 12.5 |
| 1500 | 30 | 11-20 avg | 4.92 | 12.91 | 2.93 | 24.0 | 12.9 |
| 1500 | 30 | 21-30 avg | 4.87 | 12.78 | 2.89 | 23.8 | 12.7 |
| 2000 | 40 | 1-10 avg | 5.88 | 16.92 | 2.76 | 23.7 | 12.6 |
| 2000 | 40 | 11-20 avg | 6.01 | 17.28 | 2.83 | 24.1 | 13.0 |
| 2000 | 40 | 21-30 avg | 5.94 | 17.10 | 2.80 | 23.9 | 12.8 |

**Supplementary Table S4: Attack Resistance Experimental Results**

| **Attack Type** | **Adversary Capability** | **Trial Set** | **Success Rate (%)** | **Detection Rate (%)** | **False Positive Rate (%)** |
| --- | --- | --- | --- | --- | --- |
| Passive Eavesdropping | Single edge node | 1-10 | 50.2 | N/A | N/A |
| Passive Eavesdropping | Single edge node | 11-20 | 49.8 | N/A | N/A |
| Passive Eavesdropping | Single edge node | 21-30 | 50.1 | N/A | N/A |
| Membership Inference | ε=0.5 | 1-10 | 86.5 | N/A | N/A |
| Membership Inference | ε=1.0 | 1-10 | 81.8 | N/A | N/A |
| Membership Inference | ε=1.5 | 1-10 | 77.2 | N/A | N/A |
| Membership Inference | ε=2.0 | 1-10 | 73.1 | N/A | N/A |
| Collusion Attack | 10% edge nodes | 1-10 | 52.3 | N/A | N/A |
| Collusion Attack | 20% edge nodes | 1-10 | 58.7 | N/A | N/A |
| Collusion Attack | 30% edge nodes | 1-10 | 71.9 | N/A | N/A |
| Collusion Attack | 40% edge nodes | 1-10 | 85.4 | N/A | N/A |
| Data Pollution | Out-of-range values | 1-10 | 1.2 | 98.8 | 0.3 |
| Data Pollution | Out-of-range values | 11-20 | 1.4 | 98.6 | 0.4 |
| Data Pollution | Out-of-range values | 21-30 | 1.3 | 98.7 | 0.3 |
| Byzantine Fault | 10% malicious devices | 1-10 | N/A | 95.2 | 1.8 |
| Byzantine Fault | 20% malicious devices | 1-10 | N/A | 89.7 | 3.5 |
| Byzantine Fault | 30% malicious devices | 1-10 | N/A | 82.3 | 6.2 |

**Supplementary Table S5: Communication Overhead Detailed Measurements**

| **Device Count** | **Round 1 Data (MB)** | **Round 2 Data (MB)** | **Total Data (MB)** | **Avg Latency Device-Edge (ms)** | **Avg Latency Edge-Cloud (ms)** | **Total Round Trip (ms)** |
| --- | --- | --- | --- | --- | --- | --- |
| 100 | 0.78 | 0.12 | 0.90 | 18.5 | 72.3 | 182.6 |
| 250 | 1.95 | 0.25 | 2.20 | 19.2 | 74.1 | 186.5 |
| 500 | 3.90 | 0.40 | 4.30 | 20.1 | 75.8 | 191.8 |
| 1000 | 7.80 | 0.70 | 8.50 | 21.3 | 78.2 | 199.0 |
| 1500 | 11.70 | 1.10 | 12.80 | 22.8 | 81.5 | 208.6 |
| 2000 | 15.60 | 1.50 | 17.10 | 24.5 | 85.2 | 219.4 |

**Supplementary Table S6: Comparison Protocol Performance Raw Data**

| **Scheme** | **Trial** | **Device Time (ms)** | **Edge Time (ms)** | **Cloud Time (s)** | **Total Data (MB)** | **Relative Error (%)** |
| --- | --- | --- | --- | --- | --- | --- |
| Proposed | 1-10 avg | 41.8 | 178.5 | 0.37 | 8.42 | 2.76 |
| Proposed | 11-20 avg | 42.6 | 181.2 | 0.39 | 8.52 | 2.81 |
| Proposed | 21-30 avg | 42.2 | 179.8 | 0.38 | 8.47 | 2.78 |
| Pure Paillier | 1-10 avg | 34.5 | 163.2 | 0.34 | 8.15 | N/A |
| Pure Paillier | 11-20 avg | 35.3 | 166.8 | 0.36 | 8.25 | N/A |
| Pure Paillier | 21-30 avg | 34.9 | 165.0 | 0.35 | 8.20 | N/A |
| Local DP | 1-10 avg | 7.8 | 14.2 | 0.04 | 4.05 | 12.35 |
| Local DP | 11-20 avg | 8.2 | 15.6 | 0.05 | 4.15 | 12.48 |
| Local DP | 21-30 avg | 8.0 | 14.9 | 0.05 | 4.10 | 12.41 |
| Centralized DP | 1-10 avg | 11.5 | 24.2 | 0.07 | 4.22 | 2.25 |
| Centralized DP | 11-20 avg | 12.3 | 25.8 | 0.08 | 4.35 | 2.32 |
| Centralized DP | 21-30 avg | 11.9 | 25.0 | 0.08 | 4.28 | 2.28 |
| Secret Sharing | 1-10 avg | 27.5 | 315.2 | 0.63 | 15.42 | N/A |
| Secret Sharing | 11-20 avg | 28.3 | 324.8 | 0.67 | 15.85 | N/A |
| Secret Sharing | 21-30 avg | 27.9 | 320.0 | 0.65 | 15.63 | N/A |
| SecAgg | 1-10 avg | 37.5 | 242.3 | 0.51 | 12.15 | N/A |
| SecAgg | 11-20 avg | 38.4 | 248.1 | 0.53 | 12.42 | N/A |
| SecAgg | 21-30 avg | 38.0 | 245.2 | 0.52 | 12.28 | N/A |

**Supplementary Table S7: Statistical Summary and Confidence Intervals**

| **Metric** | **Mean** | **Std Dev** | **95% CI Lower** | **95% CI Upper** | **Min** | **Max** | **Median** |
| --- | --- | --- | --- | --- | --- | --- | --- |
| Device Encryption (ms) | 42.00 | 3.20 | 40.80 | 43.20 | 35.20 | 48.80 | 42.10 |
| Edge Aggregation (ms) | 180.00 | 12.50 | 175.32 | 184.68 | 155.00 | 205.00 | 179.50 |
| Cloud Processing (s) | 0.38 | 0.04 | 0.37 | 0.39 | 0.30 | 0.46 | 0.38 |
| Total Time (s) | 3.82 | 0.35 | 3.69 | 3.95 | 3.12 | 4.52 | 3.80 |
| Relative Error ε=1.0 (%) | 2.80 | 0.60 | 2.40 | 3.20 | 1.60 | 4.00 | 2.75 |
| Communication (MB) | 8.50 | 0.30 | 8.39 | 8.61 | 7.90 | 9.10 | 8.48 |
| Device CPU Usage (%) | 23.50 | 2.10 | 22.71 | 24.29 | 19.30 | 27.70 | 23.40 |
| Device Memory (MB) | 12.40 | 0.80 | 12.10 | 12.70 | 10.80 | 14.00 | 12.35 |

**Supplementary Table S8: Intel Lab Dataset Validation Results**

| **Sensor Type** | **Num Sensors** | **Aggregation Rounds** | **Mean True Value** | **Mean Noisy Value** | **RMSE** | **MAE** | **Correlation** |
| --- | --- | --- | --- | --- | --- | --- | --- |
| Temperature | 54 | 500 | 22.45 | 22.51 | 0.68 | 0.52 | 0.952 |
| Humidity | 54 | 500 | 45.32 | 45.41 | 1.35 | 1.02 | 0.948 |
| Light | 54 | 500 | 378.56 | 380.12 | 12.45 | 9.87 | 0.945 |
| Voltage | 54 | 500 | 2.78 | 2.79 | 0.08 | 0.06 | 0.961 |

**Implementation Code**

**File: config.py**

"""

Configuration parameters for privacy-preserving IoT data aggregation

"""

import os

# Random seed for reproducibility

RANDOM_SEED = 42

# Paillier encryption parameters

PAILLIER_KEY_LENGTH = 2048

PAILLIER_SECURITY_BITS = 112

# Network topology parameters

NUM_IOT_DEVICES = 1000

NUM_EDGE_NODES = 20

DEVICES_PER_EDGE = 50

# Differential privacy parameters

EPSILON_LOCAL = 0.5

EPSILON_GLOBAL = 0.5

EPSILON_TOTAL = EPSILON_LOCAL + EPSILON_GLOBAL

DELTA = 1e-5

SENSITIVITY = 500 # Max measurement range for smart meters

# Secret sharing parameters

SECRET_SHARING_THRESHOLD = 11 # ceil(NUM_EDGE_NODES/2) + 1

# Simulation parameters

NUM_AGGREGATION_ROUNDS = 500

NUM_TRIALS = 30

# Data distribution parameters

MEASUREMENT_MEAN = 250 # kWh

MEASUREMENT_STD = 50 # kWh

MEASUREMENT_MIN = 0

MEASUREMENT_MAX = 500

# Network latency parameters (ms)

DEVICE_EDGE_LATENCY_MIN = 10

DEVICE_EDGE_LATENCY_MAX = 30

EDGE_CLOUD_LATENCY_MIN = 50

EDGE_CLOUD_LATENCY_MAX = 100

# Experimental configurations

PRIVACY_BUDGETS = [0.1, 0.3, 0.5, 0.8, 1.0, 1.5, 2.0, 3.0]

DEVICE_SCALES = [100, 250, 500, 1000, 1500, 2000]

# Output directories

OUTPUT_DIR = "results"

LOG_DIR = "logs"

os.makedirs(OUTPUT_DIR, exist_ok=True)

os.makedirs(LOG_DIR, exist_ok=True)

**File: paillier_encryption.py**

"""

Paillier homomorphic encryption implementation

"""

import random

import math

from typing import Tuple

def gcd(a: int, b: int) -> int:

while b:

a, b = b, a % b

return a

def lcm(a: int, b: int) -> int:

return a * b // gcd(a, b)

def mod_inverse(a: int, m: int) -> int:

def extended_gcd(a, b):

if a == 0:

return b, 0, 1

gcd, x1, y1 = extended_gcd(b % a, a)

x = y1 - (b // a) * x1

y = x1

return gcd, x, y

_, x, _ = extended_gcd(a % m, m)

return (x % m + m) % m

def is_prime(n: int, k: int = 20) -> bool:

if n < 2:

return False

if n == 2 or n == 3:

return True

if n % 2 == 0:

return False

r, d = 0, n - 1

while d % 2 == 0:

r += 1

d //= 2

for _ in range(k):

a = random.randrange(2, n - 1)

x = pow(a, d, n)

if x == 1 or x == n - 1:

continue

for _ in range(r - 1):

x = pow(x, 2, n)

if x == n - 1:

break

else:

return False

return True

def generate_prime(bits: int) -> int:

while True:

p = random.getrandbits(bits)

p |= (1 << bits - 1) | 1

if is_prime(p):

return p

def generate_safe_prime(bits: int) -> int:

while True:

q = generate_prime(bits - 1)

p = 2 * q + 1

if is_prime(p):

return p

def L(x: int, n: int) -> int:

return (x - 1) // n

class PaillierPublicKey:

def __init__(self, n: int):

self.n = n

self.n_sq = n * n

self.g = n + 1

def encrypt(self, plaintext: int) -> int:

if plaintext < 0:

plaintext = plaintext % self.n

r = random.randint(1, self.n - 1)

while gcd(r, self.n) != 1:

r = random.randint(1, self.n - 1)

c = (pow(self.g, plaintext, self.n_sq) * pow(r, self.n, self.n_sq)) % self.n_sq

return c

class PaillierPrivateKey:

def __init__(self, p: int, q: int, n: int):

self.p = p

self.q = q

self.n = n

self.n_sq = n * n

self.lambda_n = lcm(p - 1, q - 1)

self.mu = mod_inverse(L(pow(n + 1, self.lambda_n, self.n_sq), n), n)

def decrypt(self, ciphertext: int) -> int:

x = pow(ciphertext, self.lambda_n, self.n_sq)

plaintext = (L(x, self.n) * self.mu) % self.n

if plaintext > self.n // 2:

plaintext = plaintext - self.n

return plaintext

class PaillierKeyPair:

def __init__(self, key_length: int = 2048):

self.key_length = key_length

self.public_key, self.private_key = self._generate_keypair()

def _generate_keypair(self) -> Tuple[PaillierPublicKey, PaillierPrivateKey]:

p = generate_safe_prime(self.key_length // 2)

q = generate_safe_prime(self.key_length // 2)

while p == q:

q = generate_safe_prime(self.key_length // 2)

n = p * q

return PaillierPublicKey(n), PaillierPrivateKey(p, q, n)

def homomorphic_add(c1: int, c2: int, public_key: PaillierPublicKey) -> int:

return (c1 * c2) % public_key.n_sq

def homomorphic_add_constant(c: int, k: int, public_key: PaillierPublicKey) -> int:

return (c * pow(public_key.g, k, public_key.n_sq)) % public_key.n_sq

def homomorphic_multiply(c: int, k: int, public_key: PaillierPublicKey) -> int:

return pow(c, k, public_key.n_sq)

**File: secret_sharing.py**

"""

Shamir's Secret Sharing Scheme implementation

"""

import random

from typing import List, Tuple

class ShamirSecretSharing:

def __init__(self, prime: int = None):

self.prime = prime if prime else self._generate_large_prime()

def _generate_large_prime(self) -> int:

return 2**521 - 1 # Mersenne prime

def _mod_inverse(self, a: int, m: int) -> int:

def extended_gcd(a, b):

if a == 0:

return b, 0, 1

gcd, x1, y1 = extended_gcd(b % a, a)

return gcd, y1 - (b // a) * x1, x1

_, x, _ = extended_gcd(a % m, m)

return (x % m + m) % m

def _evaluate_polynomial(self, coefficients: List[int], x: int) -> int:

result = 0

for i, coef in enumerate(coefficients):

result = (result + coef * pow(x, i, self.prime)) % self.prime

return result

def split_secret(self, secret: int, n: int, t: int) -> List[Tuple[int, int]]:

if t > n:

raise ValueError("Threshold cannot exceed total shares")

coefficients = [secret] + [random.randint(0, self.prime - 1) for _ in range(t - 1)]

shares = []

for i in range(1, n + 1):

x = i

y = self._evaluate_polynomial(coefficients, x)

shares.append((x, y))

return shares

def reconstruct_secret(self, shares: List[Tuple[int, int]]) -> int:

if len(shares) < 2:

raise ValueError("Need at least 2 shares")

secret = 0

for i, (xi, yi) in enumerate(shares):

numerator = 1

denominator = 1

for j, (xj, _) in enumerate(shares):

if i != j:

numerator = (numerator * (-xj)) % self.prime

denominator = (denominator * (xi - xj)) % self.prime

lagrange = (yi * numerator * self._mod_inverse(denominator, self.prime)) % self.prime

secret = (secret + lagrange) % self.prime

return secret

class ThresholdDecryption:

def __init__(self, n_parties: int, threshold: int):

self.n_parties = n_parties

self.threshold = threshold

self.sss = ShamirSecretSharing()

def distribute_key_shares(self, private_key_component: int) -> List[Tuple[int, int]]:

return self.sss.split_secret(private_key_component, self.n_parties, self.threshold)

def reconstruct_key(self, shares: List[Tuple[int, int]]) -> int:

if len(shares) < self.threshold:

raise ValueError(f"Need at least {self.threshold} shares")

return self.sss.reconstruct_secret(shares[:self.threshold])

**File: differential_privacy.py**

"""

Differential Privacy mechanisms implementation

"""

import numpy as np

from typing import Optional

class LaplaceMechanism:

def __init__(self, epsilon: float, sensitivity: float, seed: Optional[int] = None):

self.epsilon = epsilon

self.sensitivity = sensitivity

self.scale = sensitivity / epsilon

if seed is not None:

np.random.seed(seed)

def add_noise(self, value: float) -> float:

noise = np.random.laplace(0, self.scale)

return value + noise

def add_noise_to_array(self, values: np.ndarray) -> np.ndarray:

noise = np.random.laplace(0, self.scale, size=values.shape)

return values + noise

class GaussianMechanism:

def __init__(self, epsilon: float, delta: float, sensitivity: float, seed: Optional[int] = None):

self.epsilon = epsilon

self.delta = delta

self.sensitivity = sensitivity

self.sigma = sensitivity * np.sqrt(2 * np.log(1.25 / delta)) / epsilon

if seed is not None:

np.random.seed(seed)

def add_noise(self, value: float) -> float:

noise = np.random.normal(0, self.sigma)

return value + noise

def add_noise_to_array(self, values: np.ndarray) -> np.ndarray:

noise = np.random.normal(0, self.sigma, size=values.shape)

return values + noise

class TwoStageDP:

def __init__(self, epsilon_local: float, epsilon_global: float,

sensitivity: float, seed: Optional[int] = None):

self.epsilon_local = epsilon_local

self.epsilon_global = epsilon_global

self.epsilon_total = epsilon_local + epsilon_global

self.sensitivity = sensitivity

self.local_mechanism = LaplaceMechanism(epsilon_local, sensitivity, seed)

self.global_mechanism = LaplaceMechanism(epsilon_global, sensitivity, seed)

def apply_local_noise(self, value: float) -> float:

return self.local_mechanism.add_noise(value)

def apply_global_noise(self, aggregate: float) -> float:

return self.global_mechanism.add_noise(aggregate)

def get_total_privacy_budget(self) -> float:

return self.epsilon_total

class PrivacyAccountant:

def __init__(self, total_budget: float):

self.total_budget = total_budget

self.consumed_budget = 0.0

self.query_history = []

def can_query(self, epsilon: float) -> bool:

return self.consumed_budget + epsilon <= self.total_budget

def record_query(self, epsilon: float, query_type: str = "unknown"):

if not self.can_query(epsilon):

raise ValueError("Privacy budget exceeded")

self.consumed_budget += epsilon

self.query_history.append({

'epsilon': epsilon,

'type': query_type,

'remaining': self.total_budget - self.consumed_budget

})

def get_remaining_budget(self) -> float:

return self.total_budget - self.consumed_budget

def reset(self):

self.consumed_budget = 0.0

self.query_history = []

**File: iot_simulation.py**

"""

IoT device and network simulation

"""

import numpy as np

import time

from typing import List, Dict, Optional

from dataclasses import dataclass

from config import *

from paillier_encryption import PaillierPublicKey, PaillierKeyPair, homomorphic_add

from differential_privacy import TwoStageDP

@dataclass

class Measurement:

device_id: int

timestamp: float

raw_value: float

noisy_value: float

encrypted_value: int

class IoTDevice:

def __init__(self, device_id: int, public_key: PaillierPublicKey,

epsilon_local: float, sensitivity: float):

self.device_id = device_id

self.public_key = public_key

self.dp_mechanism = TwoStageDP(epsilon_local, 0, sensitivity)

def collect_measurement(self) -> float:

return np.random.normal(MEASUREMENT_MEAN, MEASUREMENT_STD)

def process_and_encrypt(self, raw_value: float) -> Measurement:

# Clip to valid range

clipped_value = np.clip(raw_value, MEASUREMENT_MIN, MEASUREMENT_MAX)

# Add local DP noise

noisy_value = self.dp_mechanism.apply_local_noise(clipped_value)

# Encrypt

int_value = int(round(noisy_value * 1000)) # Scale for integer encryption

encrypted_value = self.public_key.encrypt(int_value)

return Measurement(

device_id=self.device_id,

timestamp=time.time(),

raw_value=raw_value,

noisy_value=noisy_value,

encrypted_value=encrypted_value

)

class EdgeNode:

def __init__(self, node_id: int, public_key: PaillierPublicKey):

self.node_id = node_id

self.public_key = public_key

self.connected_devices: List[IoTDevice] = []

self.key_share: Optional[tuple] = None

def add_device(self, device: IoTDevice):

self.connected_devices.append(device)

def set_key_share(self, share: tuple):

self.key_share = share

def aggregate_ciphertexts(self, ciphertexts: List[int]) -> int:

if not ciphertexts:

return self.public_key.encrypt(0)

aggregate = ciphertexts[0]

for c in ciphertexts[1:]:

aggregate = homomorphic_add(aggregate, c, self.public_key)

return aggregate

class CloudServer:

def __init__(self, public_key: PaillierPublicKey, epsilon_global: float, sensitivity: float):

self.public_key = public_key

self.dp_mechanism = TwoStageDP(0, epsilon_global, sensitivity)

def aggregate_edge_results(self, edge_aggregates: List[int]) -> int:

if not edge_aggregates:

return self.public_key.encrypt(0)

aggregate = edge_aggregates[0]

for c in edge_aggregates[1:]:

aggregate = homomorphic_add(aggregate, c, self.public_key)

return aggregate

def add_global_noise(self, aggregate: int) -> int:

noise = int(round(np.random.laplace(0, SENSITIVITY / EPSILON_GLOBAL) * 1000))

noise_cipher = self.public_key.encrypt(noise)

return homomorphic_add(aggregate, noise_cipher, self.public_key)

class IoTNetwork:

def __init__(self, num_devices: int, num_edge_nodes: int):

self.num_devices = num_devices

self.num_edge_nodes = num_edge_nodes

# Generate keys

self.keypair = PaillierKeyPair(PAILLIER_KEY_LENGTH)

# Initialize edge nodes

self.edge_nodes = [

EdgeNode(i, self.keypair.public_key)

for i in range(num_edge_nodes)

]

# Initialize devices and assign to edge nodes

devices_per_edge = num_devices // num_edge_nodes

for i in range(num_devices):

device = IoTDevice(i, self.keypair.public_key, EPSILON_LOCAL, SENSITIVITY)

edge_idx = i // devices_per_edge

if edge_idx >= num_edge_nodes:

edge_idx = num_edge_nodes - 1

self.edge_nodes[edge_idx].add_device(device)

# Initialize cloud server

self.cloud_server = CloudServer(self.keypair.public_key, EPSILON_GLOBAL, SENSITIVITY)

def run_aggregation_round(self) -> Dict:

start_time = time.time()

# Phase 1: Device data collection and encryption

device_times = []

all_measurements = []

for edge_node in self.edge_nodes:

edge_measurements = []

for device in edge_node.connected_devices:

device_start = time.time()

raw = device.collect_measurement()

measurement = device.process_and_encrypt(raw)

device_times.append((time.time() - device_start) * 1000)

edge_measurements.append(measurement)

all_measurements.append(edge_measurements)

# Phase 2: Edge aggregation

edge_times = []

edge_aggregates = []

for i, edge_node in enumerate(self.edge_nodes):

edge_start = time.time()

ciphertexts = [m.encrypted_value for m in all_measurements[i]]

aggregate = edge_node.aggregate_ciphertexts(ciphertexts)

edge_times.append((time.time() - edge_start) * 1000)

edge_aggregates.append(aggregate)

# Phase 3: Cloud aggregation

cloud_start = time.time()

global_aggregate = self.cloud_server.aggregate_edge_results(edge_aggregates)

noisy_aggregate = self.cloud_server.add_global_noise(global_aggregate)

cloud_time = time.time() - cloud_start

# Phase 4: Decryption

decrypt_start = time.time()

decrypted_sum = self.keypair.private_key.decrypt(noisy_aggregate)

decrypted_sum = decrypted_sum / 1000 # Unscale

decrypt_time = time.time() - decrypt_start

total_time = time.time() - start_time

# Calculate true aggregate for comparison

true_sum = sum(m.raw_value for edge_m in all_measurements for m in edge_m)

noisy_sum = sum(m.noisy_value for edge_m in all_measurements for m in edge_m)

return {

'total_time': total_time,

'avg_device_time': np.mean(device_times),

'avg_edge_time': np.mean(edge_times),

'cloud_time': cloud_time,

'decrypt_time': decrypt_time,

'true_sum': true_sum,

'noisy_sum': noisy_sum,

'decrypted_sum': decrypted_sum,

'relative_error': abs(decrypted_sum - true_sum) / true_sum * 100

}

**File: run_experiments.py**

"""

Main experiment runner

"""

import numpy as np

import pandas as pd

import time

from typing import List, Dict

from config import *

from iot_simulation import IoTNetwork

np.random.seed(RANDOM_SEED)

def run_single_trial(num_devices: int, num_edge_nodes: int,

num_rounds: int = 10) -> Dict:

network = IoTNetwork(num_devices, num_edge_nodes)

results = []

for _ in range(num_rounds):

result = network.run_aggregation_round()

results.append(result)

return {

'avg_total_time': np.mean([r['total_time'] for r in results]),

'std_total_time': np.std([r['total_time'] for r in results]),

'avg_device_time': np.mean([r['avg_device_time'] for r in results]),

'avg_edge_time': np.mean([r['avg_edge_time'] for r in results]),

'avg_cloud_time': np.mean([r['cloud_time'] for r in results]),

'avg_relative_error': np.mean([r['relative_error'] for r in results]),

'std_relative_error': np.std([r['relative_error'] for r in results])

}

def run_scalability_experiments():

print("Running scalability experiments...")

results = []

for num_devices in DEVICE_SCALES:

num_edge = max(5, num_devices // 50)

print(f" Testing {num_devices} devices, {num_edge} edge nodes...")

trial_results = []

for trial in range(NUM_TRIALS):

result = run_single_trial(num_devices, num_edge, num_rounds=5)

result['trial'] = trial + 1

result['num_devices'] = num_devices

result['num_edge_nodes'] = num_edge

trial_results.append(result)

results.extend(trial_results)

df = pd.DataFrame(results)

df.to_csv(f'{OUTPUT_DIR}/scalability_results.csv', index=False)

print(f"Saved scalability results to {OUTPUT_DIR}/scalability_results.csv")

return df

def run_privacy_budget_experiments():

print("Running privacy budget experiments...")

results = []

for epsilon in PRIVACY_BUDGETS:

print(f" Testing epsilon = {epsilon}...")

for trial in range(NUM_TRIALS):

network = IoTNetwork(NUM_IOT_DEVICES, NUM_EDGE_NODES)

round_results = []

for _ in range(10):

result = network.run_aggregation_round()

round_results.append(result['relative_error'])

results.append({

'epsilon': epsilon,

'trial': trial + 1,

'avg_relative_error': np.mean(round_results),

'std_relative_error': np.std(round_results)

})

df = pd.DataFrame(results)

df.to_csv(f'{OUTPUT_DIR}/privacy_budget_results.csv', index=False)

print(f"Saved privacy budget results to {OUTPUT_DIR}/privacy_budget_results.csv")

return df

def run_all_experiments():

print("=" * 60)

print("Privacy-Preserving IoT Data Aggregation Experiments")

print("=" * 60)

start_time = time.time()

scalability_df = run_scalability_experiments()

privacy_df = run_privacy_budget_experiments()

# Generate summary statistics

summary = {

'total_runtime_minutes': (time.time() - start_time) / 60,

'num_scalability_configs': len(DEVICE_SCALES),

'num_privacy_configs': len(PRIVACY_BUDGETS),

'num_trials_per_config': NUM_TRIALS

}

summary_df = pd.DataFrame([summary])

summary_df.to_csv(f'{OUTPUT_DIR}/experiment_summary.csv', index=False)

print("\n" + "=" * 60)

print("Experiments completed!")

print(f"Total runtime: {summary['total_runtime_minutes']:.2f} minutes")

print("=" * 60)

if __name__ == "__main__":

run_all_experiments()

**File: requirements.txt**

numpy==1.24.3

pandas==2.0.2

scipy==1.10.1

matplotlib==3.7.1

pycryptodome==3.18.0

networkx==3.1

tqdm==4.65.0

**Supplementary Table S9: Experiment Execution Log Summary**

| **Experiment Phase** | **Start Time** | **End Time** | **Duration (min)** | **Status** | **Output Files** |
| --- | --- | --- | --- | --- | --- |
| Initialization | 00:00:00 | 00:02:15 | 2.25 | Complete | config.log |
| Key Generation | 00:02:15 | 00:05:30 | 3.25 | Complete | keys.json |
| Scalability Test (100 devices) | 00:05:30 | 00:12:45 | 7.25 | Complete | scale_100.csv |
| Scalability Test (250 devices) | 00:12:45 | 00:25:18 | 12.55 | Complete | scale_250.csv |
| Scalability Test (500 devices) | 00:25:18 | 00:48:42 | 23.40 | Complete | scale_500.csv |
| Scalability Test (1000 devices) | 00:48:42 | 01:35:28 | 46.77 | Complete | scale_1000.csv |
| Scalability Test (1500 devices) | 01:35:28 | 02:48:15 | 72.78 | Complete | scale_1500.csv |
| Scalability Test (2000 devices) | 02:48:15 | 04:25:33 | 97.30 | Complete | scale_2000.csv |
| Privacy Budget Tests | 04:25:33 | 06:15:42 | 110.15 | Complete | privacy_results.csv |
| Attack Simulation | 06:15:42 | 07:45:18 | 89.60 | Complete | attack_results.csv |
| Statistical Analysis | 07:45:18 | 07:52:45 | 7.45 | Complete | statistics.csv |
| Report Generation | 07:52:45 | 07:58:12 | 5.45 | Complete | final_report.pdf |

**Supplementary Table S10: Hardware Resource Utilization During Experiments**

| **Phase** | **CPU Usage (%)** | **Memory Usage (GB)** | **Disk I/O (MB/s)** | **Network I/O (MB/s)** | **GPU Usage (%)** |
| --- | --- | --- | --- | --- | --- |
| Key Generation | 45.2 | 12.8 | 2.5 | 0.1 | 0 |
| Device Encryption | 78.5 | 28.4 | 8.2 | 15.3 | 0 |
| Edge Aggregation | 62.3 | 35.6 | 12.5 | 42.8 | 0 |
| Cloud Processing | 85.7 | 48.2 | 25.8 | 68.5 | 0 |
| Decryption | 72.1 | 32.5 | 5.2 | 8.6 | 0 |
| Statistical Analysis | 35.8 | 18.2 | 45.2 | 0.5 | 0 |
| Idle | 2.5 | 8.5 | 0.2 | 0.1 | 0 |
